# Genetic Adaptation of Siberian Larch (*Larix sibirica* Ledeb.) to High Altitudes

**DOI:** 10.3390/ijms24054530

**Published:** 2023-02-25

**Authors:** Serafima V. Novikova, Vadim V. Sharov, Natalia V. Oreshkova, Evgeniy P. Simonov, Konstantin V. Krutovsky

**Affiliations:** 1 Laboratory of Genomic Research and Biotechnology, Federal Research Center “Krasnoyarsk Science Center of the Siberian Branch of the Russian Academy of Sciences”, 660036 Krasnoyarsk, Russia; 2 Laboratory of Forest Genomics, Genome Research and Education Center, Institute of Fundamental Biology and Biotechnology, Siberian Federal University, 660041 Krasnoyarsk, Russia; 3 Department of High-Performance Computing, Institute of Space and Information Technologies, Siberian Federal University, 660074 Krasnoyarsk, Russia; 4 Tauber Bioinformatics Research Center, University of Haifa, Haifa 3498838, Israel; 5 Laboratory of Forest Genetics and Selection, V. N. Sukachev Institute of Forest, Siberian Branch of Russian Academy of Sciences, 660036 Krasnoyarsk, Russia; 6 Department of Genomics and Bioinformatics, Institute of Fundamental Biology and Biotechnology, Siberian Federal University, 660041 Krasnoyarsk, Russia; 7 Laboratory of Evolutionary Trophology, A. N. Severtsov Institute of Ecology and Evolution, Russian Academy of Sciences, 119071 Moscow, Russia; 8 Department of Forest Genetics and Forest Tree Breeding, Georg-August University of Göttingen, 37077 Göttingen, Germany; 9 Center for Integrated Breeding Research, George-August University of Göttingen, 37075 Göttingen, Germany; 10 Laboratory of Population Genetics, N. I. Vavilov Institute of General Genetics, Russian Academy of Sciences, 119333 Moscow, Russia; 11 Scientific and Methodological Center, G. F. Morozov Voronezh State University of Forestry and Technologies, 394087 Voronezh, Russia

**Keywords:** adaptation, admixture, altitudes, BayeScEnv, bioclimatic variables, conifer, ddRADseq, *F*_ST_, *Larix sibirica*, LFMM, outlier, PCAdapt, RDA, Siberian larch, SNPs

## Abstract

Forest trees growing in high altitude conditions offer a convenient model for studying adaptation processes. They are subject to a whole range of adverse factors that are likely to cause local adaptation and related genetic changes. Siberian larch (*Larix sibirica* Ledeb.), whose distribution covers different altitudes, makes it possible to directly compare lowland with highland populations. This paper presents for the first time the results of studying the genetic differentiation of Siberian larch populations, presumably associated with adaptation to the altitudinal gradient of climatic conditions, based on a joint analysis of altitude and six other bioclimatic variables, together with a large number of genetic markers, single nucleotide polymorphisms (SNPs), obtained from double digest restriction-site-associated DNA sequencing (ddRADseq). In total, 25,143 SNPs were genotyped in 231 trees. In addition, a dataset of 761 supposedly selectively neutral SNPs was assembled by selecting SNPs located outside coding regions in the Siberian larch genome and mapped to different contigs. The analysis using four different methods (PCAdapt, LFMM, BayeScEnv and RDA) revealed 550 outlier SNPs, including 207 SNPs whose variation was significantly correlated with the variation of some of environmental factors and presumably associated with local adaptation, including 67 SNPs that correlated with altitude based on either LFMM or BayeScEnv and 23 SNPs based on both of them. Twenty SNPs were found in the coding regions of genes, and 16 of them represented non-synonymous nucleotide substitutions. They are located in genes involved in the processes of macromolecular cell metabolism and organic biosynthesis associated with reproduction and development, as well as organismal response to stress. Among these 20 SNPs, nine were possibly associated with altitude, but only one of them was identified as associated with altitude by all four methods used in the study, a nonsynonymous SNP in scaffold_31130 in position 28092, a gene encoding a cell membrane protein with uncertain function. Among the studied populations, at least two main groups (clusters), the Altai populations and all others, were significantly genetically different according to the admixture analysis based on any of the three SNP datasets as follows: 761 supposedly selectively neutral SNPs, all 25,143 SNPs and 550 adaptive SNPs. In general, according to the AMOVA results, genetic differentiation between transects or regions or between population samples was relatively low, although statistically significant, based on 761 neutral SNPs (*F*_ST_ = 0.036) and all 25,143 SNPs (*F*_ST_ = 0.017). Meanwhile, the differentiation based on 550 adaptive SNPs was much higher (*F*_ST_ = 0.218). The data showed a relatively weak but highly significant linear correlation between genetic and geographic distances (*r* = 0.206, *p* = 0.001).

## 1. Introduction

Siberian larch (*Larix sibirica* Ledeb.) is one of the key conifer species of Siberian boreal forests, playing a very important ecological and economical role. Siberian larch has a high level of phenotypic variation, the genetic mechanisms of which are still poorly understood. The genetic study of this species is hampered by the huge size of the genome, ~12 Gbp [1], which was almost completely sequenced, assembled and annotated [2,3].

At the moment, to study the adaptation of organisms to growing conditions, bioclimatic and stress factors and their impact, high-throughput sequencing of genomic DNA regions associated with restriction sites (RADseq) is widely used [4]. The approach allows researchers to genotype thousands of markers, mainly single nucleotide polymorphisms (SNPs), more or less uniformly and randomly representing the majority of the genome. The high efficiency of this method and its relatively low cost, reproducibility and high fidelity make it possible to analyze hundreds of samples in a short time.

At the same time, landscape genomics methods are widely used. They are based on a relatively new approach that simultaneously analyzes variation of a large number of genes and environmental factors to detect genes whose variation is under selection and presumably associated with adaptation to environmental factors, the so-called candidate adaptive genes [5,6,7]. The rapid growth of studies that use landscape genomics methods over the past two decades can be explained by increased interest in the ecological and evolutionary consequences of current environmental changes, such as loss and fragmentation of habitats [8,9] or human-caused climate change [10]. In particular, understanding and predicting the consequences of ongoing environmental changes can be considered one of the main contemporary research tasks because humans cause significant changes in the environment and associated loss of biodiversity. Thanks also to modern technological advances, landscape genomics has great potential now to contribute to such studies, so it is not surprising that landscape genomics studies have grown exponentially since 2003, including in tree conservation [11].

Two main strategies have been developed to identify loci under selection, the variation of which may have an adaptive value (or loci linked to those). The first strategy is to search for loci with striking values of genetic differentiation that cannot be explained by only random selectively neutral processes such as genetic drift and isolation (the so-called *F*_ST_-outlier test) [12], while the other strategy is based on the search for significant associations between the variation of genetic markers and the variation of environmental factors (genotype– or genome–environment associations—GEAs) [13].

The first approach is based on the assumption that positive selection for different alleles in populations living in different ecological environments increases the divergence between them to a level that cannot be explained by selectively neutral processes such as genetic drift or isolation, while stabilizing selection preserves the degree of divergence at a level statistically lower than can be expected by chance [14]. The second approach suggests that the allele frequencies of loci associated with the variability of certain adaptive traits (growth rate, flowering time, resistance to diseases and stress, etc.) or involved in the process of adaptation to specific environmental conditions (such as temperature, humidity, atmospheric pressure, length of the growing season, etc.) should correlate with the variability of these adaptive traits and environmental factors or have striking differences in allele frequencies between geographic regions [15].

For example, Eckert et al. [16] found a significant correlation between genetic variation of drought-related loci in loblolly pine (*Pinus taeda* L.) and climatic variables. De Kort et al. [17] studied regional climate adaptation of drought-sensitive black alder (*Alnus glutinosa* L.) across Europe and reported significant associations between variation of several loci of this tree species and temperature and latitude. Zheng et al. [18] discovered two specific regions in the Tibetan poplar (*Populus szechuanica* var. *tibetica*) genome associated with altitude and response to the solar radiation level, and gained insight into the genetic mechanisms underlying the adaptation to highlands in plants.

Plants growing in different altitude conditions are subjected to different environmental factors and are a convenient model for studying of the adaptation process. Highland plants are subject to a whole range of adverse factors: high-intensity exposure to solar radiation and wind, low atmospheric pressure, low temperatures, sharp fluctuations in daily and seasonal temperatures, humidity, and a short growing season. These climatic factors act as major forces in the selection of fitness-enhancing variants from the gene pool and hence stimulate local adaptation and genetic differentiation. Plant species whose habitat spans different altitudes (from sea level to above 2000 m) are of the greatest interest, allowing direct comparisons of lowland and highland populations.

This study was aimed at identifying signs of local adaptation in the Siberian larch populations using genome-wide genotyping and landscape genomics approaches. We studied genetic differentiation of Siberian larch populations associated with adaptation to the altitudinal gradient of climatic conditions based on joint analysis of six bioclimatic variables and double digest restriction-site-associated DNA sequencing (ddRADseq) data. Specifically, we analyzed Siberian larch population structure, genetic diversity and genetic traits of adaptation to growing conditions within the Altai-Sayan mountain system in southeastern Siberia.

## 2. Results

### 2.1. Environmental Variables

**Figure 1:**
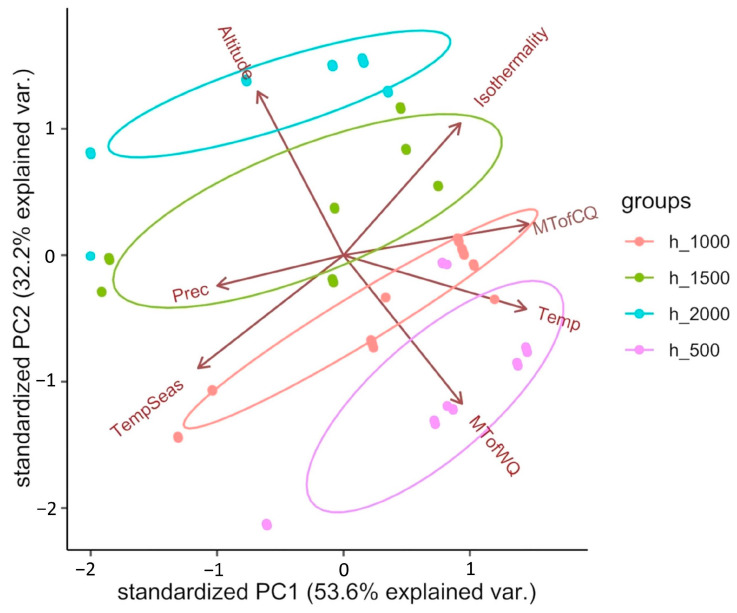
Principal component analysis (PCA) plot of seven environmental factors.

PCA was performed to pre-test the relationship between climate variables and altitude at the collection sites. The first principle component (PC1) explained 53.6% of the variation primarily related to the average annual precipitation (PREC) and the average temperature of the coldest quarter (MTofCQ)—lower PREC and higher MTofCQ correspond to positive PC1 values. The second principle component (PC2) explained 32.2% of the variation primarily related to the altitude (ALT) and average temperature of the warmest quarter (MTofWQ)—higher ALT and lower MTofWQ correspond to positive PC2 values (Figure 1). The correlation between the values of climate variables and the first two principal components PC1 and PC2 is presented in Table 1.

**Figure 2:**
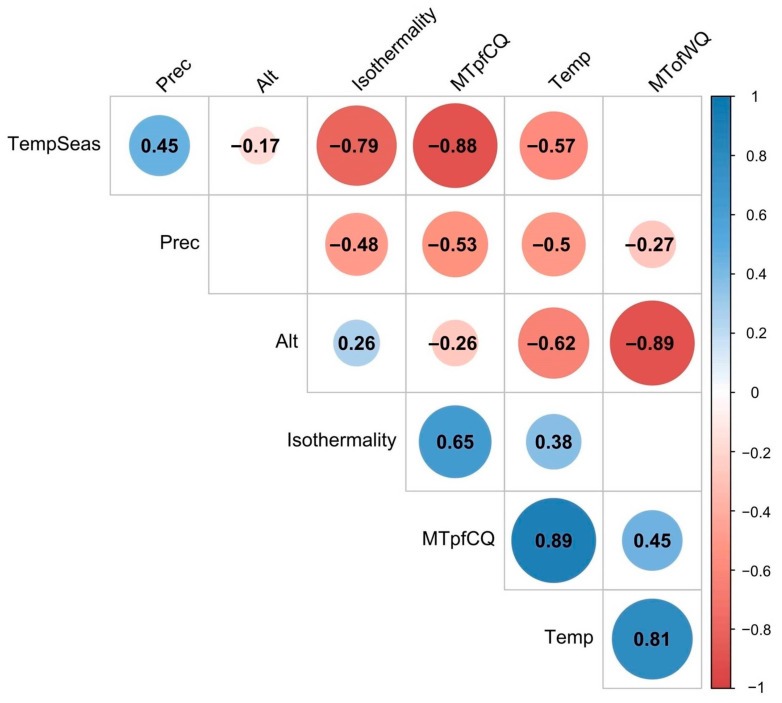
Pairwise Pearson’s correlation coefficients (*r*) between six bioclimatic variables (Temp, Isothermality, TempSeas, MTofWQ, MTofCQ, and Prec; see Table 1) and altitude (ALT). The graph shows only significant correlation values (*p* < 0.01). For better visualization, significance is also displayed with the color and size of the circle around the correlation value.

Pairwise Pearson’s correlation coefficients (*r*) between six bioclimatic variables and altitude (ALT) are shown in Figure 2.

The average annual temperature (TEMP) was significantly positively correlated with the average temperature of the warmest (MTofWQ) and coldest (MTofCQ) quarters with correlation coefficients of 0.81 (*p* ≪ 0.001) and 0.89 (*p* ≪ 0.001), respectively. The altitude (ALT) and the MTofWQ, as well as the temperature seasonality (TEMPSEAS) and the MTofCQ, were also significantly but negatively correlated with each other (*r* = −0.89 and −0.88, *p* ≪ 0.001, respectively).

### 2.2. SNP Dataset

More than 3.2 billion up to 100 bp long single-end reads were obtained in total for 250 trees. After primary processing and quality filtering, about 3.1 billion reads were selected for further analysis in 231 trees, with an average of 13.5 million reads per sample (ranging from 1.6 to 49.2 million) and an average sequence length of 85 bp (ranging from 32 to 92 bp). About 97% of the reads for each sample on average were successfully mapped to the reference Siberian larch genome [2]. The mapping results are presented in Table S1.

Data for 19 trees were completely removed from further analysis due to the small number of reads (≤1 million) and the insufficient level of mapping to the reference genome. Finally, 19,743 loci containing 25,143 biallelic SNPs were selected for genotyping of 231 trees in total (Data S1).

In addition, in order to infer a potential population genetic structure that resulted due to random selectively neutral factors such as genetic drift and isolation, a dataset of 761 supposedly selectively neutral SNPs was assembled by selecting SNPs located outside coding regions in the genome and mapped to different contigs. In addition, a dataset of 550 supposedly adaptive SNPs was assembled as described below based on the outlier SNPs and SNPs whose variation correlated with altitude and/or bioclimatic variables (Table S2) to compare the results for this “adaptive” SNP dataset with the results for the “selectively neutral” SNP dataset.

### 2.3. Detection of SNPs Associated with Environmental Variables and Outliers

SNPs whose level of variation and differentiation cannot be explained by selective-neutral processes are likely to be under selection and possibly involved in local biological adaptation. We used several of the most efficient population genetic approaches to find such candidate adaptive SNPs.

**Figure 3:**
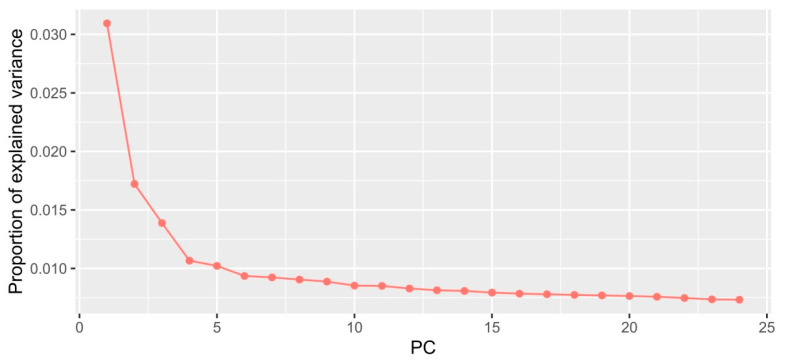
Proportion of variance explained by each PC.

The PCAdapt [19] program was used to test how much each SNP was associated with population structure, assuming that outlier SNPs were indicative of local adaptation. First, we conducted the PCA on SNP genotypes to find the PCs that best explained the genetic structure across individuals. The graph in Figure 3 demonstrates that PC1 and PC2 (*K* = 2) explained the most genetic variance; for that reason, they were retained for further analysis.

**Figure 4:**
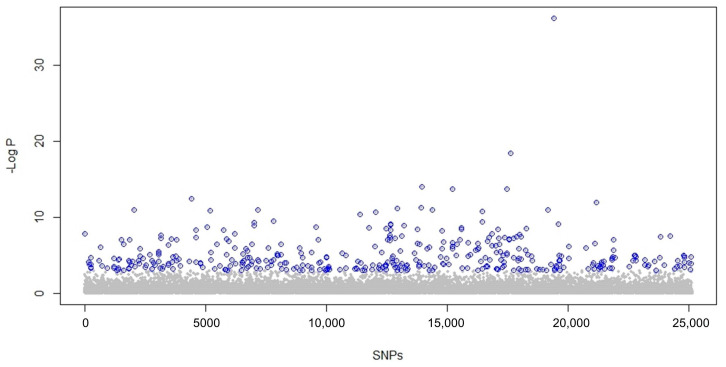
Distribution of the statistical significance score for each marker based on their *p*-values obtained in PCAdapt; 423 outlier SNPs are highlighted in blue after applying the false discovery rate (FDR) cut-off with a *q*-value < 0.05.

Then, all SNPs were regressed against the retained ordination axes, and outlier SNPs were selected based on their significant correlation with these axes. The Manhattan plot in Figure 4 shows the statistical significance score for each SNP. In total, 423 outlier SNPs were identified using the false discovery rate (FDR) cut-off with a *q*-value < 0.05.

Using the latent factor mixed model program LFMM2 [20], 40 SNPs were found whose variation correlated with the compositional predictor PC1, mainly representing the variation of such bioclimatic factors as the average annual precipitation (PREC) and the average temperature of the coldest quarter (MTofCQ), and variation of 49 SNPs correlated with altitude (ALT) (FDR *q*-value < 0.05), three of which were common for both predictors, PC1 and ALT.

Similarly, when using the BayeScEnv program [21], 94 SNPs were found whose variation correlated with the PC1 compositional predictor, including 41 SNPs that also correlated with altitude (ALT) (FDR *q*-value < 0.05).

The correlation of environmental factors with the first three redundancy analysis (RDA) axes is presented in Table 2.

**Figure 5:**
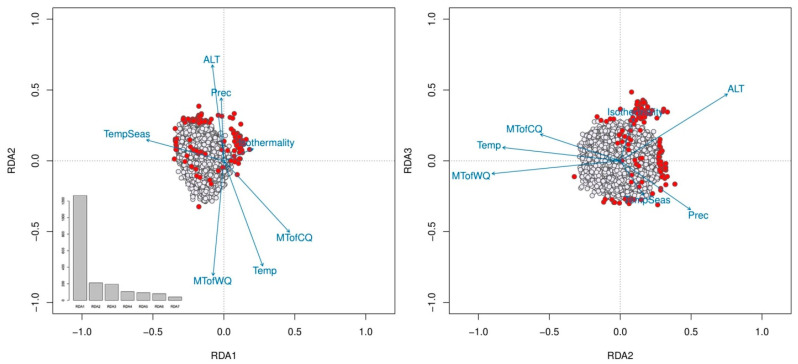
The eigenvalues of the first three RDA axes and projection of SNPs on RDA1 and RDA2 (**left plot**) and RDA2 and RDA3 (**right plot**). Significant SNPs are highlighted in red.

The eigenvalues of the first three RDA axes and projection of SNPs on them are presented in Figure 5. In total, 158 significant SNPs were found, including 46 that were significant across multiple RDA axes (two-tailed *p*-value = 0.0027). Alt, Prec and Isotermality were most closely related to the SNPs found (Figure 5).

**Figure 6:**
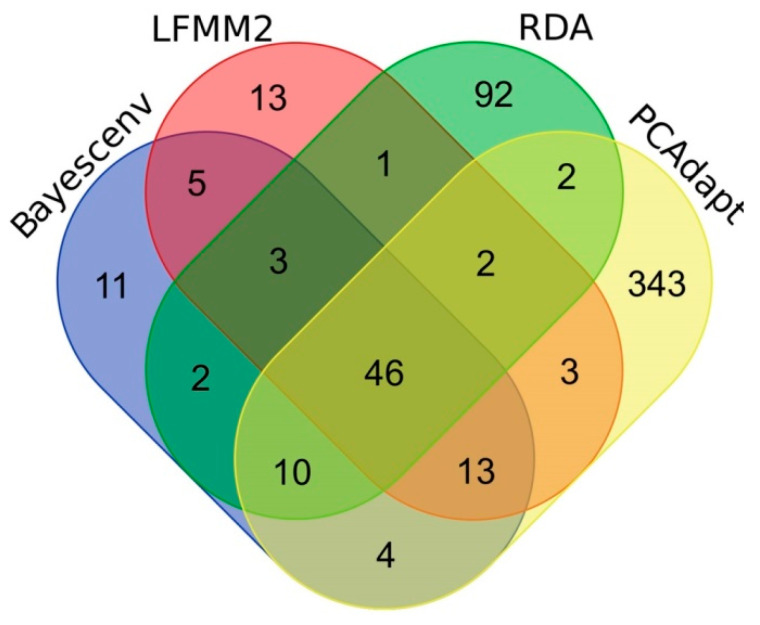
Venn diagram summarizing the results of the search for significant outlier SNPs using the four methods, LFMM, BayeScEnv, RDA and PCAdapt.

In total, when combining all four methods, 550 unique significant outlier SNPs were revealed, including 49 that correlated with environmental factors and were common for all three GEA methods (LFMM, BayeScEnv and RDA) and 46 for all four methods (previous three plus PCAdapt) (Figure 6). Among the 550 SNPs, 67 SNPs correlated with altitude based on LFMM and/or BayeScEnv, and 23 of them - based on both LFMM and BayeScEnv. Among 46 SNPs that correlated with environmental factors and identified also by PCAdapt, 43 correlated with altitude based on LFMM and/or BayeScEnv.

### 2.4. Population Genetic Variation, Structure and Differentiation

The summary of genetic variation parameters for each of the 24 population samples based on 25,143 SNPs is presented in Table 3, and based on 761 neutral and 550 adaptive outlier SNPs, in Table S3.

**Figure 7:**
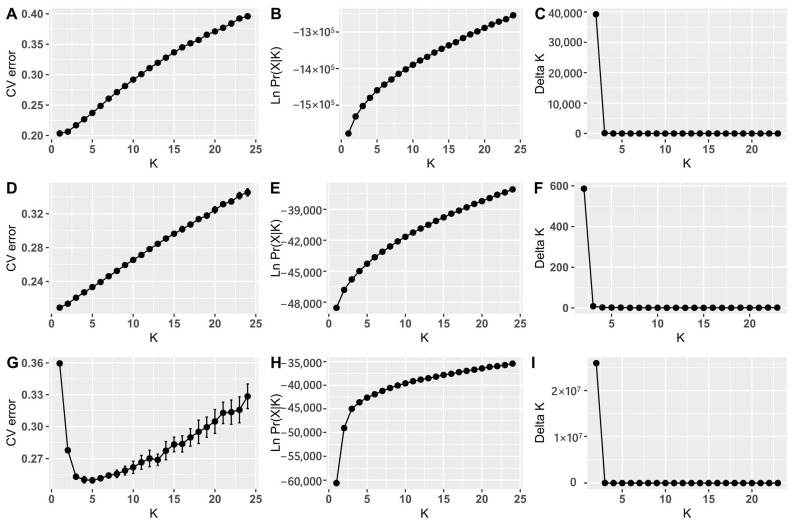
Graphs displaying the results of analysis of probable number of clusters *K*. Cross-validation prediction error, log likelihood and Δ*K* values for each *K* based on all 25,143 SNPs (**A**–**C**), 761 neutral SNPs (**D**–**F**) and 550 adaptive SNPs (**G**–**I**).

The most probable number of subpopulation clusters (*K*) was searched using three different datasets of SNPs—all 25,143 SNPs, 761 supposedly neutral SNPs and 550 adaptive SNPs whose variation is supposedly under selection (described below in detail)—by checking the value of the parameter *K* (from 1 to 24) in a computer simulation with 20 repetitions (iterations) for each number of *K*. Various methods for selecting *K* showed that the most probable number of clusters was *K* = 2 (Figure 7).

**Figure 8:**
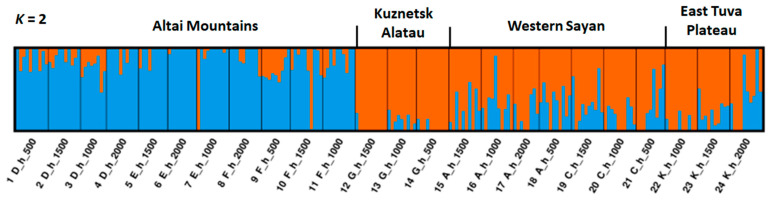
Admixture of the two clusters (*K* = 2) indicated by two different colors (Q-values) in individual Siberian larch trees, representing 24 samples collected at different altitudes in four geographic regions and based on 761 neutral SNPs.

A plot demonstrating the admixture of each of the two clusters to individual trees (Q-values) is presented in Figure 8. It can be seen that the trees collected in the Altai Mountains represent a distinct cluster that is genetically mostly different from trees collected in other regions. Admixture plots at different *K* (from *K* = 1 to *K* = 6), based on three different datasets of SNPs and sorted differently according to their geographic origin and altitude, respectively, are presented in Figure S1.

**Figure 9:**
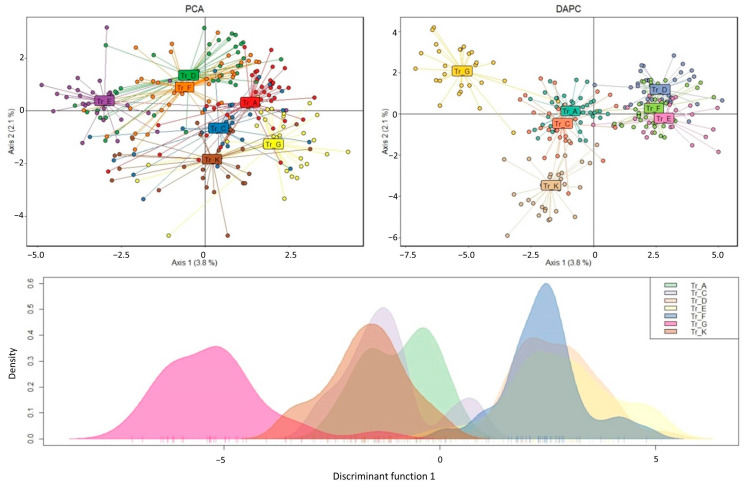
Results of principal component analysis (PCA, **top left**) and discriminant analysis of principal components (DAPC, **top right**), and plot of sample density along the first discriminant function (**bottom**).

PCA and DAPC were also performed to identify the population structure based on 761 neutral SNPs. It was revealed that the studied samples did not form pronounced, unambiguous clusters; however, we could infer the presence of two or three conditional clusters: the samples belonging to transects A, C and K formed one cluster, the samples of transects D, E and F formed the second and transect G, according to the DAPC results, occupied a separate position (Figure 9).

**Figure 10:**
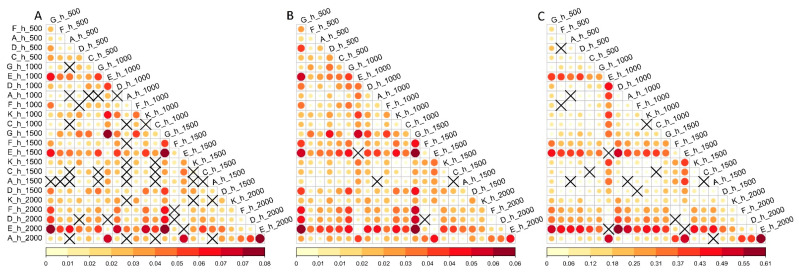
Pairwise *F*_ST_ fixation indices calculated between 24 population samples based on 761 neutral SNPs (**A**), all 25,143 SNPs (**B**) and 550 adaptive SNPs (**C**). Significant values are highlighted by color (*p* < 0.05), with the shade and size of the circles reflecting the magnitude. Cross marks depict insignificant values.

Genetic differentiation was measured using the *F*_ST_ parameter [22] for the following four groupings by dividing the population samples: (1) into two clusters according to the results of the admixture algorithm [23] (*K* = 2), when geographic transects D, E and F were in one cluster (Altai Mountains) and A, C, G and K were in another (*F*_ST_ = 0.018); (2) into three clusters (*K* = 3), in which transects D, E and F formed one cluster (Altai Mountains) and transects A, C (Western Sayan Mountains) and K (East Tuva Highlands) the second, while transect G (Kuznetsk Alatau) formed an independent third cluster (*F*_ST_ = 0.013 between G and the group of A, C and K; *F*_ST_ = 0.039 between G and the group of D, E and F; *F*_ST_ = 0.0153 between the group of A, C and K and the group of D, E and F); (3) into seven geographic transect clusters (*K* = 7, mean *F*_ST_ = 0.023), with the lowest *F*_ST_ between transects D and F (0.004) and the highest between G and E (0.061); (4) into 24 clusters corresponding the 24 samples, respectively (mean *F*_ST_ = 0.028, with a minimum value of 0.0006 between samples A_h_500 and A_h_1000 and a maximum of 0.083 between E_h_2000 and G_h_1500, Figure 10).

Based on three different SNP datasets, a hierarchical AMOVA was also carried out by partitioning the total genetic variance into among 7 geographic transects (regions), among 3–4 population samples within transects, and within and among all 24 population samples, then calculating Wright’s fixation indices (*F*-indices) for each hierarchical level (Table 4). They were relatively low (0.015–0.036) but significant based on neutral or all SNPs and much higher and highly significant based on adaptive SNPs (0.149–0.218, *p* ≪ 0.001).

**Figure 11:**
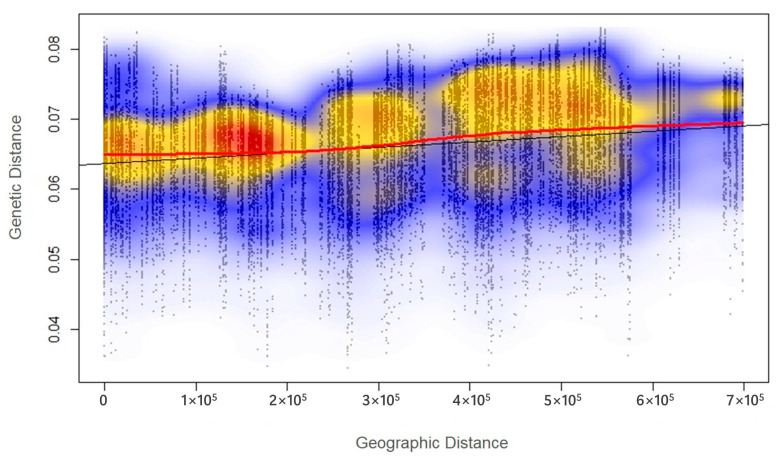
Graph showing linear relationship between genetic and geographic distances based on the Mantel test and all 25,143 SNPs (*r* = 0.206, *p* = 0.001). The different colors represent different densities of genetic and geographic distance correlation values (red—high density, blue—low density), the smoothed local mean (red line) and the regression (black line).

Geographically limited distribution can shape the genetic structure of a population and lead to a correlation between genetic and geographic distance, called isolation by distance. The Mantel test was carried out using all 25,143 SNPs to find such correlation, and its results revealed a relatively weak, but highly significant (*r* = 0.206, *p* = 0.001) linear relationship between genetic distance and geographic distance (Figure 11).

### 2.5. SNP Annotation

Out of 550 significant SNPs, 61 were located in 49 scaffolds that included the annotated genes: 20 SNPs were located within the coding regions of genes, and 41 in intergenic regions, including 18 at a distance of less than 10 Kbp from genes. Regarding the 67 SNPs associated with altitude, four were located in three scaffolds that included the annotated genes: three were located in intergenic regions, including one SNP at a distance of less than 10 Kbp from genes, and one SNP was located in scaffold_31130 (in position 28092) within a coding region of gene LS_31130-0.0 (Table S4).

**Figure 12:**
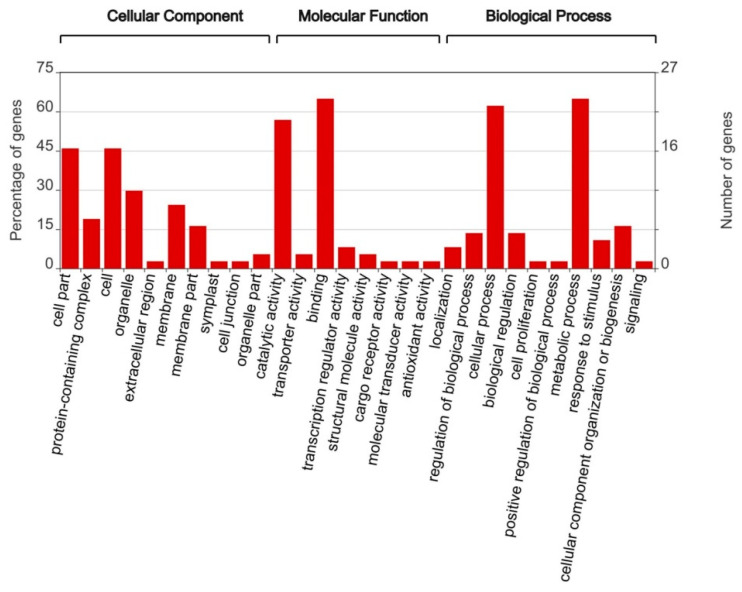
GO (Gene Ontology) annotation of genes in scaffolds with significant outlier SNPs.

Cellular components where the functioning of the products of these genes was localized included a wide range of membrane complexes (Figure 12 and Table S4).

The main biological processes in which they are involved are the processes of macromolecular cell metabolism and organic biosynthesis, including metabolism of nitrogenous and aromatic compounds, phosphorus, nitrogenous bases, carbohydrates and proteins, as well as related processes of regulation of gene expression (Figure 12 and Figure S2).

Among the 61 SNPs, 16 represented non-synonymous single nucleotide substitutions that potentially affect the function of the corresponding proteins and are of the most interest for further study.

A BLAST search in the NCBI GenBank [24] for sequences homologs to the scaffolds with significant SNPs but without annotated genes found highly similar sequences representing some regions of the mitochondrial and chloroplast genomes of gymnosperms. Alignment of these sequences to the organelle genomes of the Siberian larch allowed us to identify 85 SNPs of organelle origin, but all of them were located in noncoding regions of the mitochondrial genome. Some of them may represent regulatory regions and require additional detailed analysis in a separate study.

## 3. Discussion

The results of the presented genome-wide analysis of the structure and genetic variation of natural populations of Siberian larch are generally consistent with previous conclusions about the relatively weak selectively neutral structure of closely located populations of conifers, including larch [25,26], explained mostly by intensive gene flow [27]. PCAdapt demonstrated that none of the PCs had eigenvalues greater than random. However, this does not mean that there is no genetic structure in the data; it just means that the structure is not particularly strong and/or cannot be easily partitioned into discrete clusters.

Most of the genetic variance (approximately 96%) when studying the neutral, adaptive and all SNPs in the 24 population samples was within samples. However, it is interesting to note that all mean parameters of genetic variation were higher for the SNP dataset based on 550 adaptive SNPs compared to the other two datasets, except for the number of private alleles (*PrA*), which was the highest for the SNP dataset based on all SNPs (Table 5). This indirectly confirms that 550 adaptive SNPs might indeed include SNPs under selection.

In general, genetic differentiation between transects or regions (*F*_CT_), between population samples within transects (*F*_SC_) and between all population samples (*F*_ST_) was relatively low, although statistically significant, based on 761 neutral SNPs and all 25,143 SNPs, as expected for conifers with large continuous populations and high gene flow. However, the differentiation based on 550 adaptive SNPs was much higher, which verifies their role in local adaptation leading to higher differentiation.

The studied population samples could be divided into at least two main groups (clusters)—the Altai populations and all other populations. That was in agreement with their altitudinal–latitudinal location, which likely leads to strong isolation of the Altai populations from all other populations. The data showed a relatively weak but significant (*r* = 0.206, *p* = 0.001) correlation between genetic and geographic distances assuming that isolation by distance plays an important role in genetic differentiation between these populations.

Searching for candidate adaptive markers using four different methods yielded a dataset of 550 supposedly adaptive SNPs (Table S2). Based on annotation, 20 of them were located in exonic, 41 in intergenic and 489 in nongenic regions (Table S4). Among these 550 SNPs, 67 SNPs were likely associated with altitude based on at least one of two methods, LFMM or BayeScEnv. Nine of them were located in six genes, but only one was identified as associated with altitude by all four methods used in the study, a nonsynonymous SNP in scaffold_31130 in position 28,092, representing a gene encoding a cell membrane protein with uncertain function (Table S4).

The following brief description of the protein products of the identified genes, in the coding regions of which we found SNPs that significantly correlated with environmental factors, may indicate the functional role that these genes play in genetic adaptation to environmental factors.

Synonymous SNP scaffold_9849_59355 and nonsynonymous SNP scaffold_9849_59395 were outliers based on PCAdapt but were not selected by the three GEA methods (LFMM, BayeScEnv and RDA). They were located in the gene LS_9849-0.1 (based on annotation presented in [3]) that encodes late embryogenesis abundant protein (LEA) D-34 (Table S4). LEA genes are expressed in seeds, seedlings, roots and other organs throughout the developmental stage. In response to environmental stressors, plants accumulate high levels of LEA proteins. They have been suggested to have a variety of functions including protecting cellular structures from the effects of water loss and desiccation, protecting proteins from stress-induced damage, sequestering ions and folding denatured proteins. LEA proteins can also act as chaperone proteins to resist cellular damage [28].

Nonsynonymous SNP scaffold_36255_2146 was associated with synthetic predictor PC1 in the LFMM analysis and located in the gene LS_36255-0.2 [3] that encodes nucleoredoxin 1 (NRX1) (Table S4). In plant cells, NRX1 oxidoreductase protects antioxidant enzymes such as catalase from ROS-induced oxidation. It was shown that NRX1 can play an important role in *Arabidopsis thaliana* (Col-0 ecotype), directly regulating the ability of cells to detoxify H_2_O_2_ [29] and thereby protecting plant cells from environmentally induced oxidative stress.

Nonsynonymous SNPs scaffold_73031_9433 and scaffold_73031_9438 were associated with all environmental predictors and located in the gene LS_73031-0.1 [3] that encodes At1g67340-like F-box protein (Table S4). The F-box is a protein motif of about 50 amino acids that functions as a protein–protein interaction site. F-box proteins were first characterized as components of SCF ubiquitin ligase complexes, in which they bind substrates for ubiquitin-mediated proteolysis. F-box proteins have been found to function in protein complexes other than SCF in various cellular functions [30].

Nonsynonymous SNP scaffold_118661_524 was an outlier based on PCAdapt but was not selected by the three GEA methods. It is located in the gene LS_118661-0.1 [3] that encodes protein EXORDIUM-like 3 (EXO) (Table S4). This protein has been identified as a potential mediator of brassinosteroid (BR)-promoted growth [31]. The EXO gene is required for cell expansion in leaves. Gene expression patterns and growth assays suggest that EXO mediates BR-induced leaf growth. EXO is thought to be involved in the signaling process that coordinates BR responses with environmental or developmental cues.

Nonsynonymous SNP scaffold_3984510_7337 was an outlier based on PCAdapt but was not selected by the three GEA methods. It is located in the gene LS_3984510-0.1 [3] that encodes conserved oligomeric Golgi complex subunit 1-like (COG1) (Table S4). COG maintains the correct structure and function of the Golgi complex during retrograde vesicle transport. In *Arabidopsis thaliana*, the COG complex functions during cell growth, reproduction and other processes including direct interaction with the components of the secretion system. Recent experiments have revealed the protective role of the COG complex in plants, including plant–pathogen interactions [32].

Nonsynonymous SNP scaffold_4015301_864 was associated with all environmental predictors and located in the gene LS_4015301-0.0 [3] that encodes fatty acid acyl-CoA reductase 4 isoform X2 (FAR4) (Table S4). This protein catalyzes the reduction in saturated but not unsaturated C16 or C18 fatty acyl-CoA to fatty alcohols. In a recent study of *Arabidopsis* [33], FAR4 (along with FAR1 and FAR5) was named as responsible for the formation of primary fatty alcohols associated with suberin. Suberin is a protective biopolyester composed of ferulic acid, glycerol and aliphatic moieties.

Nonsynonymous SNP scaffold_4023983_5884 was an outlier based on PCAdapt but was not selected by the three GEA methods. It is located in the gene LS_4023983-0.1 [3] that encodes adapter protein complex 2 subunit 1 (AP2A1) (Table S4). AP2 forms the central part of clathrin-dependent endocytosis by simultaneously binding to carrier proteins, plasma membrane lipids and clathrin. It was shown that in *Arabidopsis*, AP2 is involved in the endocytosis of the BRASSINOSTEROID INSENSITIVE1 (BRI1) receptor [34], which is part of the signaling cascade of brassinosteroids, phytohormones with strong growth-stimulating activity involved in the regulation of many biological processes, including resistance to abiotic stresses and developmental processes such as flowering time, fertility and pollen development.

Nonsynonymous SNP scaffold_4033175_6319 was an outlier based on PCAdapt but was not selected by the three GEA methods. It is located in the gene LS_4033175-0.1 [3] that encodes cellulose synthase-like protein E6 (CSL) (Table S4), a representative of a subfamily of enzymes closely related to cellulose synthases, which in some plant species, are involved in the biosynthesis of cellulose and various polymers of β-glycans [35].

Synonymous SNP scaffold_4078980_3679 was an outlier based on PCAdapt but was not selected by the three GEA methods. It is located in the gene LS_4078980-0.1 [3] that encodes ISWI chromatin-remodeling complex ATPase (Table S4), which regulates transcription of coding and noncoding RNA by mobilizing nucleosomes and controlling the length of linker DNA that separates nucleosomes [36].

Nonsynonymous SNP scaffold_5133697_5690 was an outlier based on PCAdapt but was not selected by the three GEA methods. It is located in the gene LS_5133697-0.1 [3] that encodes A1 PLIP2 glycerolipid phospholipase (Table S4). It was shown that overexpression of PLIP2 strongly reduces plant growth and leads to accumulation of the bioactive form of jasmonate and related oxylipins [37]. PLIP2 in *Arabidopsis* provides a link between the ABA-mediated response to abiotic stress and oxylipin signaling.

Nonsynonymous SNP scaffold_5135911_1098 was an outlier based on PCAdapt but was not selected by the three GEA methods. It is located in the gene LS_5135911-0.1 [3] that encodes plant intracellular Ras-group-related LRR protein 1 (PIRL1) (Table S4). PIRLs are distinct from the larger, well-characterized classes of plant LRR proteins. Characterization of mutants with T-DNA insertion showed that PIRL1 plays an important role in the early stages of pollen development [38].

Nonsynonymous SNP scaffold_5188799_2951 was an outlier based on PCAdapt but was not selected by the three GEA methods. It is located in the gene LS_5188799-0.1 [3] that encodes oligopeptide transporter 7 (OPT7) (Table S4). Peptide transport involves the translocation of peptides (2–6 amino acid long residues) across the cell membrane in an energy-dependent manner. The identification of several OPTs in *Arabidopsis* suggests that they may play different functional roles [39].

Among the 550 candidate adaptive SNPs found in this study, 41 were located in the intergenic regions of the genome. Some of these SNPs are likely to be located in regulatory regions and affect gene expression, which requires additional research.

Previously, Zheng et al. [18] revealed the presence of altitudinal adaptation in the Tibetan poplar population. Two hotspot regions of the genome were detected, one of which (four genes, chromosome 15) was associated with altitudinal variation, and the other (10 genes, chromosome 6) with response to solar radiation. Among the genes identified in this work, one gene was orthologous to At3g47110 found in *A. thaliana*; the LRR protein encoded by this gene interacts with ferric reductase defective 3 (FRD3), which is involved in citrate transport and stable development of microspores during pollen tube growth. Another gene encodes MADS-box transcription factor 47, which is involved in the formation of floral organs, in part through downregulation of the brassinosteroid signaling pathway. Phospholipid hydroperoxide glutathione peroxidase 1 (GPX) is a group of proteins that protect cells from oxidative damage caused by reactive oxygen species (ROS).

## 4. Materials and Methods

### 4.1. Plant Material and DNA Isolation

**Figure 13:**
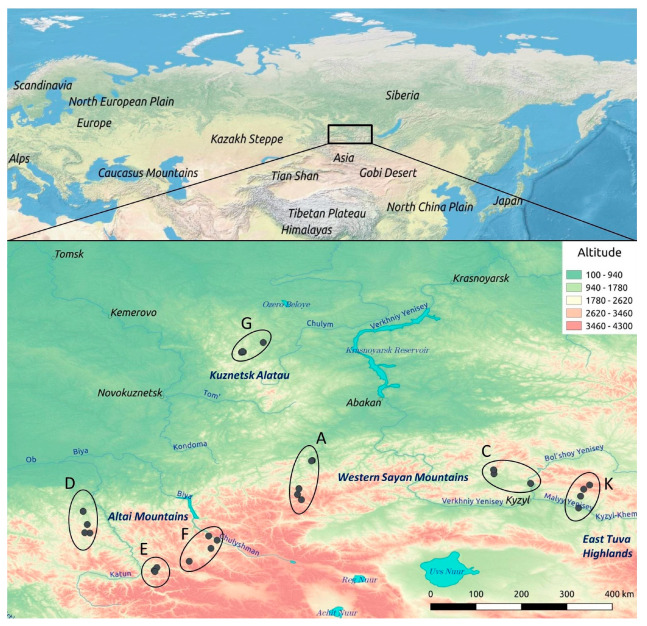
Locations of the studied samples in the Altai-Sayan region. A: Sayan Pass region, along the Abakan-Ak-Dovurak highway, south of the town of Abaza, the border of Khakassia and Tyva, the Dzhebash Ridge and the Western Sayan Mountain range; C: near the town of Turan, the border of Khakassia and Tyva, the Kurtushibinsky Ridge and the Western Sayan Mountain range; D: north of the Seminsky Pass, Chuisky Trakt, Shebalinsky District, the Republic of Altai, Seminsky Range and the Altai Mountains; E: Ongudaysky District, the Republic of Altai, Achik Pass, Bely Bom mountain, North Chuysky Ridge and the Altai Mountains; F: Ulagansky District, the Republic of Altai, western bank of the Chulyshman River, Ulagan Highlands and the Altai Mountains; G: near the village of Priiskovy, the Republic of Khakassia and Kuznetsk Alatau; K: western part of the Kaa-Khemsky District of Tyva, Academician Obruchev Ridge and the East Tuva Highlands.

Individual needle samples were collected from 250 trees (~20–100 years old) of Siberian larch along seven high-altitude transects (A, C, D, E, F, G and K) located in the Altai-Sayan region in southeastern Siberia in native unprotected area (Figure 13).

Along each transect, 3–4 population samples of 10 trees each were collected at different altitudes, where each sample corresponded approximately to 500, 1000, 1500 or 2000 m above sea level (Table 3).

DNA from the collected larch needles was isolated using the CTAB method [40]. The DNA concentration was assessed using a Qubit 2.0 fluorimeter and a Qubit dsDNA BR Assay Kit (Thermo Fisher Scientific, Waltham, MA, USA). The purity and quality of the isolated DNA were also assessed using the Implen NanoPhotometer P330 (Implen, München, Germany). High-quality DNA samples with a A260/230 ratio of ~1.8 and a concentration of 20–150 ng/µL were selected for this work.

### 4.2. Library Construction

Preparation of ddRADseq libraries was carried out according to a modified version of the protocol described in [4]. DNA samples were digested with two restriction enzymes, *EcoR*I and *Mse*I [41], selected by in silico modeling using the reference Siberian larch genome [2] and the ddRADseqTool program [42]. After treatment with restriction enzymes, barcoded adapters were ligated for each sample. Fragmented DNA with ligated adapters was purified using Agencourt AMPureXP magnetic particles (Beckman Coulter, Brea, CA, USA). Then, PCR amplification of the ligation products was carried out using high-precision Q5 High-Fidelity polymerase (New England BioLabs, Ipswich, MA, USA). The obtained PCR products of the samples were combined into pools of 60–80 samples per pool. For subsequent sequencing, 300–700 bp long fragments were isolated for each pool by cutting out a piece of gel from 2.5% agarose gel after electrophoresis corresponding to 300–700 bp long zone. DNA was extracted from the gel using the QIAquick Gel Extraction Kit (Qiagen, Hilden, Germany).

The obtained pools of ddRADseq libraries were checked for quality by capillary electrophoresis on a Bioanalyzer 2100 instrument using a High Sensitivity DNA Kit (Agilent Technologies, Santa Clara, CA, USA). The pool concentration was measured on a Qubit 2.0 fluorimeter using a Qubit dsDNA BR Assay Kit (Thermo Fisher Scientific, Waltham, MA, USA). The final check of the prepared pools before sequencing was carried out on an Agilent 2200 TapeStation System (Agilent Technologies, Santa Clara, CA, USA).

Single-end sequencing of ddRADseq libraries was performed using 100 cycles on a NovaSeq 6000 sequencer (Illumina, San Diego, CA, USA). Accordingly, the length of the reads was 100 bp, and the number of reads varied in the range of 400–500 million per transect.

### 4.3. Bioclimatic Data

In addition to the geographic coordinates and altitude measured at the collection sites for each tree, by using their exact coordinates and the R “raster” v. 3.4.5 [43] and “sp” [44] programs, individual data were obtained for the following six bioclimatic variables from the WorldClim database [45]: (1) Temp—average annual temperature, (2) Isothermality— the ratio of the average annual temperature to the average annual temperature amplitude multiplied by 100, (3) TempSeas—temperature seasonality, the amount of temperature change over a certain period based on the ratio of the standard deviation of average monthly temperatures to the average monthly temperature, (4) MTofWQ—average temperature of the warmest quarter, (5) MTofCQ—average temperature of the coldest quarter, and (6) Prec—average annual precipitation.

All values were standardized for further calculations by subtracting the arithmetic mean and dividing by the standard deviation using the basic R scale function. Genes associated with environmental variables and potentially reflecting local adaptation were detected using the LFMM2 [20] and BayeScEnv [21] programs. The first principal component (PC1) in the principal component analysis (PCA) was used as a compositional predictor for six climate variables, while the altitude variable (ALT) was analyzed separately.

### 4.4. SNP Calling

The raw sequencing data went through several steps of initial processing. The original reads were filtered and trimmed according to quality scores in the Trimmomatic-0.39 program [46] with parameters MINQUAL = 23 and MINLEN = 40. Each sequence was checked for the presence of *EcoR*I and *Mse*I restriction sites. Demultiplexing was performed based on the barcoded adapter sequences unique for each sample using the process_radtags utility included in the Stacks software [47]. Statistics after demultiplexing were collected by multiqc [48]. The average read length for the samples after processing was 85 bp. Filtered reads were aligned to the reference Siberian larch genome using the Bowtie 2 program v. 2.3 [49] in the “--local” mapping mode with default parameters and selection of uniquely aligned reads.

Alignment results were sorted, and the genomic assembly was indexed in Samtools [50]. SNP calling was performed by Gstacks utility from the Stacks software with filtering by the quality of read alignment “--min-mapq 20”. The resulting set of alignment-covered loci was subjected to several filtering steps using the Populations utility to keep only the loci that were present in at least 80% of all samples (--min-samples-overall 0.8) and in 60% of trees in each population sample (--min-samples-per-pop 0.6). The maximum allowed level of observed heterozygosity for each accepted SNP could not exceed 0.6 (--max-obs-het 0.6), the minimum minor allele frequency 0.01 and the minimum coverage 3.

The search for SNPs located in intergenic areas was carried out using the annotation of Siberian larch [3] in SNPdat [51]. Since most of the methods used in our study were sensitive to the presence of missing data, the missing allele frequencies were generated using the *k*-nearest neighbor genotype imputation method (LD-kNNi) in the TASSEL v. 5.0 program [52].

### 4.5. Detection of SNPs Associated with Environmental Variables and Outliers

The search for SNPs with striking values of genetic differentiation, which could not be explained only by selectively neutral processes, the so-called outlier genes, was carried out using four approaches: (1) PCA-based genome scans for selection using the PCAdapt v4.3.3 program [19], (2) regularized least squares estimates for latent factor mixed models (LFMM) using the LFMM2 program [20], (3) Bayesian analysis of the polynomial Dirichlet model using the BayeScEnv v. 1.1 program [21] and (4) redundancy analysis (RDA) using the vegan R package [53]. PCAdapt and LFMM2 were run with 2–5 *K* clusters. BayeScEnv is an *F*_ST_-based, genome scan method that uses environmental variables to detect local adaptation. BayeScEnv models were run separately for PC1 and ALT variables using the same parameters: 20 pilot runs with 5000 iterations, thinning interval size 10, 5000 outputted iterations and burn-in length 50,000.

### 4.6. Population Genetic Variation, Structure and Differentiation

The following R packages were used for the analysis of population genetic variation: adegenet [54], poppr [55], and vcfR [56]. For each of the 24 population samples, the following parameters were calculated: the number of private alleles (*PrA*), allelic richness (A_R_), observed (*H_o_*) and expected (*H_e_*) heterozygosity and fixation index (*F*_IS_). To reveal the population structure, we also performed principal component analysis (PCA) and discriminant analysis of principal components (DAPC) using the R ade4 package [57].

In addition, the structure of the population was studied using the admixture algorithm implemented in the AdmixPipe program [22], which estimates the maximum likelihood of suggested genetic clusters based on genotypic data. To do so, a search for the most probable number (*K*) of clusters (“subpopulations”) was carried out by checking the value of the parameter *K* from 1 to 24, with 20 repetitions for each *K*. The most probable value of *K* was chosen based on the values of the cross-validation error and the ΔK method [58] calculated using Clumpak [59].

A pairwise population genetic distance matrix for the Mantel test was built in the TASSEL program, where genetic distance was calculated as 1-IBS (identity by state) based on all 25,143 SNPs. This was used in the Mantel test to check for correlation with pairwise geographic distance using the R vegan v. 2.6-2 package. Hierarchical analysis of molecular variance (AMOVA) and calculation of pairwise *F*_ST_ coefficients based on 1000 permutations were performed using Arlequin v. 3.5.1.2 [60].

### 4.7. SNP Annotation

To analyze genomic regions in the contigs where adaptive SNPs were located, we used these contigs to search for homologs in the “nr” database of the NCBI GenBank [24]. Gene models were aligned to the base “nr” filtered for Embryophyta species using a taxonomic identifier. The search for protein domains was performed using InterProScan [61] on the EMBL-EBI server [62]. The corresponding Gene Ontology terms were obtained using Blast2GO on the OmixBox platform (https://www.biobam.com/omicsbox, accessed on 30 December 2022). The selected SNPs were annotated using SNPdat.

## 5. Conclusions

We identified several SNPs in candidate genes whose variation was associated with altitude and other bioclimatic variables, such as LEA, NRX1, F-box, EXO, COG1, FAR4, AP2A1, CSL, ISWI, PLIP2, PIRL1 and OPT7-like genes. Based on these results, it can be assumed that Siberian larch has adapted to high altitudes in part through supportive functions associated with reproduction under abiotic stress, such as chaperone protection against cellular damage, cell growth support and stimulation, stress signaling, epigenetic regulation via chromatin remodeling, etc., although more information about how these genes regulate altitude adaptation in Siberian larch must be ascertained. The results of this study will allow for a deeper understanding of the genetic mechanisms underlying the formation of adaptations in larch to various environmental conditions. Considering that many environmental factors affecting larch in high-altitude conditions can be confidently attributed to stress, this study allows us to detect important genes and SNP markers for breeding, as well as lays the foundation for creating a SNP genotyping chip for monitoring neutral and adaptive genetic variability in other larch populations. The presented data can serve as a scientific basis for optimizing nature management, developing methods for the rational use of the studied species, identifying populations with good genetic potential and conducting environmental monitoring.

## Figures and Tables

**Table 1:** Correlation coefficient values between principal components PC1 and PC2 and climate variables.

Climate Variable	PC1	PC2
Temp	**−0.467**	**−0.363**
Isothermality	**−0.378**	**0.481**
TempSeas	**0.445**	**−0.358**
MTofWQ	−0.245	**−0.713**
MTofCQ	**−0.513**	−0.009
Prec	**0.343**	−0.020

Note. Climate variables are explained in the text. In the row for each variable, numbers indicate the strength of correlation of that variable with the eigenvector of each PC. The correlation coefficients with values more than 0.3 are considered important in defining the PC and highlighted by bold font.

**Table 2:** Correlation of environmental factors with the first three RDA axes.

Environmental Factor	RDA1	RDA2	RDA3
Alt	−0.015	0.259	0.155
Temp	0.051	−0.284	0.031
Isothermality	0.039	0.032	0.099
TempSeas	−0.102	0.056	−0.079
MTofWQ	−0.014	−0.310	−0.030
MTofCQ	0.087	−0.193	0.061
Prec	−0.003	0.170	−0.113

**Table 3:** The summary of genetic variation parameters ± SE based on 25,143 SNPs for each of the 24 population samples of Siberian larch.

Region	Transect	Population Sample	*N*	*PrA*	*A_R_*	*H_o_*	*H_e_*	*F*_IS_
Western Sayan Mountain	A	A_h_500	10	5	1.257 ± 0.002	0.043 ± 0.001	0.061 ± 0.001	0.199 ± 0.004 **
		A_h_1000	10	0	1.275 ± 0.002	0.055 ± 0.001	0.062 ± 0.001	0.071 ± 0.003 **
		A_h_1500	10	0	1.299 ± 0.002	0.060 ± 0.001	0.068 ± 0.001	0.081 ± 0.003 **
		A_h_2000	8	0	1.250 ± 0.002	0.041 ± 0.001	0.060 ± 0.001	0.213 ± 0.005 **
	C	C_h_500	9	16	1.278 ± 0.002	0.054 ± 0.001	0.067 ± 0.001	0.123 ± 0.004 **
		C_h_1000	10	16	1.269 ± 0.002	0.044 ± 0.001	0.063 ± 0.001	0.207 ± 0.004 **
		C_h_1500	10	0	1.247 ± 0.002	0.046 ± 0.001	0.057 ± 0.001	0.132 ± 0.004 **
Altai Mountains	D	D_h_500	10	0	1.282 ± 0.002	0.067 ± 0.001	0.066 ± 0.001	−0.007 ± 0.002
		D_h_1000	8	0	1.284 ± 0.003	0.066 ± 0.001	0.065 ± 0.001	−0.009 ± 0.002
		D_h_1500	10	6	1.261 ± 0.002	0.061 ± 0.001	0.061 ± 0.001	0.000 ± 0.002
		D_h_2000	10	0	1.284 ± 0.002	0.066 ± 0.001	0.065 ± 0.001	−0.007 ± 0.002
	E	E_h_1000	10	5	1.276 ± 0.002	0.054 ± 0.001	0.064 ± 0.001	0.108 ± 0.003 **
		E_h_1500	9	0	1.280 ± 0.002	0.060 ± 0.001	0.066 ± 0.001	0.051 ± 0.003 **
		E_h_2000	9	8	1.271 ± 0.003	0.055 ± 0.001	0.065 ± 0.001	0.097 ± 0.004 **
	F	F_h_500	9	0	1.285 ± 0.002	0.067 ± 0.001	0.064 ± 0.001	−0.026 ± 0.002 **
		F_h_1000	10	6	1.270 ± 0.002	0.064 ± 0.001	0.062 ± 0.001	−0.015 ± 0.002 *
		F_h_1500	10	0	1.255 ± 0.002	0.058 ± 0.001	0.057 ± 0.001	−0.005 ± 0.002
		F_h_2000	10	0	1.260 ± 0.002	0.062 ± 0.001	0.059 ± 0.001	−0.026 ± 0.002 **
Kuznetsk Alatau	G	G_h_500	10	15	1.305 ± 0.002	0.065 ± 0.001	0.073 ± 0.001	0.068 ± 0.003 **
		G_h_1000	9	5	1.295 ± 0.002	0.061 ± 0.001	0.071 ± 0.001	0.091 ± 0.003 **
		G_h_1500	10	33	1.302 ± 0.002	0.065 ± 0.001	0.074 ± 0.001	0.073 ± 0.003 **
East Tuva Highlands	K	K_h_1000	10	15	1.283 ± 0.002	0.053 ± 0.001	0.067 ± 0.001	0.129 ± 0.003 **
		K_h_1500	10	9	1.300 ± 0.002	0.064 ± 0.001	0.071 ± 0.001	0.062 ± 0.003 **
		K_h_2000	10	4	1.312 ± 0.002	0.065 ± 0.001	0.072 ± 0.001	0.065 ± 0.004 **
Mean			9.6	5.958 ± 1.652	1.278 ± 0.004	0.058 ± 0.002	0.065 ± 0.001	0.070 ± 0.015

*N*—number of trees, *PrA*—number of private alleles, *A_R_*—allelic richness, *H_o_*—observed heterozygosity, *H_e_*—expected heterozygosity, *F*_IS_— fixation index; * *p* < 0.05, ** *p* < 0.001.

**Table 4:** AMOVA results for three different SNP datasets.

Source of Variation	Sum of Squares	Variance Components	Percentage Variation, %	*F*-Index
761 neutral SNPs				
Among transects	391.778	0.491	1.885	*F*_CT_ = 0.019
Among populations within transects	571.122	0.453	1.739	*F*_SC_ = 0.018
Within populations	10,693.785	25.097	96.376	*F*_ST_ = 0.036
Total	11,656.685	26.041		
All 25,143 SNPs				
Among transects	11,683.915	14.009	1.675	*F*_CT_ = 0.017
Among populations within transects	17,631.271	11.997	1.433	*F*_SC_ = 0.015
Within populations	347,430.578	810.654	96.892	*F*_ST_ = 0.017
Total	376,745.764	836.661		
550 adaptive SNPs				
Among transects	2819.564	5.896	14.854	*F*_CT_ = 0.149
Among populations within transects	1422.569	2.748	6.923	*F*_SC_ = 0.081
Within populations	13,413.821	31.048	78.223	*F*_ST_ = 0.218
Total	17,655.955	39.692		

**Table 5:** Mean genetic variation parameters for 24 population samples of Siberian larch based on three different SNP datasets (±SE).

SNP Dataset	*PrA*	*A_R_*	*H_o_*	*H_e_*	*F*_IS_
All 25,143 SNPs	5.958	1.278 ± 0.004	0.058 ± 0.002	0.065 ± 0.001	0.070 ± 0.015
761 neutral SNPs	0.375	1.282 ± 0.006	0.058 ± 0.002	0.067 ± 0.001	0.087 ± 0.017
550 adaptive SNPs	0.994	1.364 ± 0.024	0.097 ± 0.007	0.114 ± 0.008	0.113 ± 0.028

## Data Availability

The data presented in this study are all available in the article and files in the supplementary material.

## Supplementary Materials

The following supporting information can be downloaded at: https://www.mdpi.com/article/10.3390/ijms24054530/s1.
