# Supplementary material for: Genetic Adaptation of Siberian Larch (Larix sibirica Ledeb.) to High Altitudes
Source: Int J Mol Sci. 2023 Feb 25;24(5):4530. doi: 10.3390/ijms24054530 (PMC10003562; doi:10.3390/ijms24054530)
Supplement: Supplementary file 1 [file ijms-24-04530-s001.zip › Figure S1. Admixture results_revised.pdf]

based on 761 supposedly selectively neutral SNPs

samples arranged according to their geographic origin

K = 2

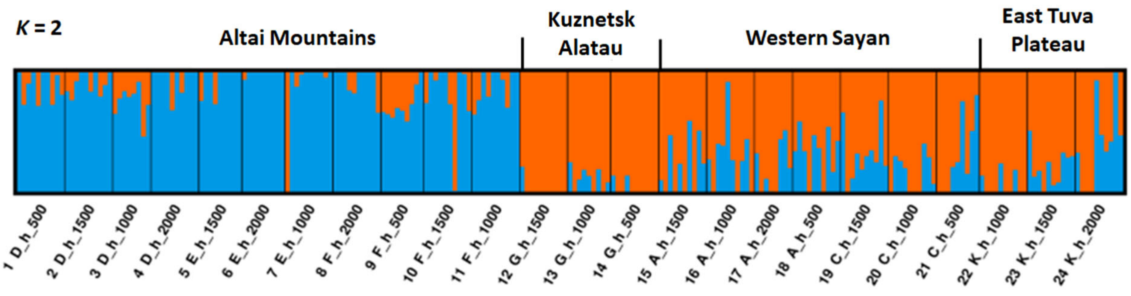

K = 3

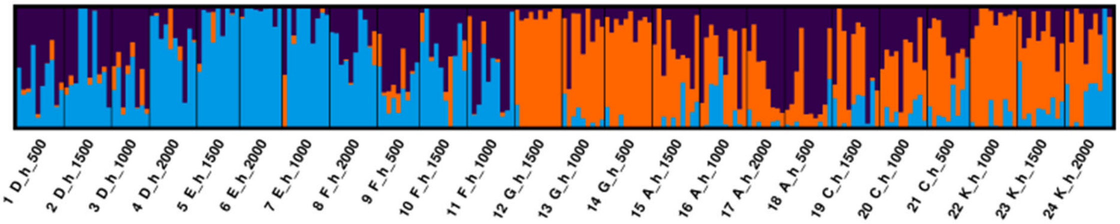

K = 4

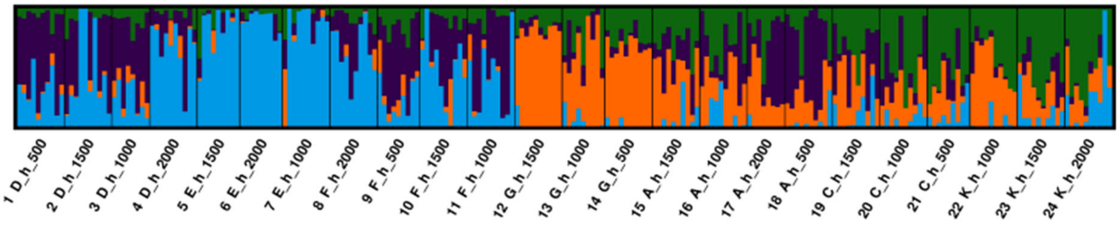

K = 5

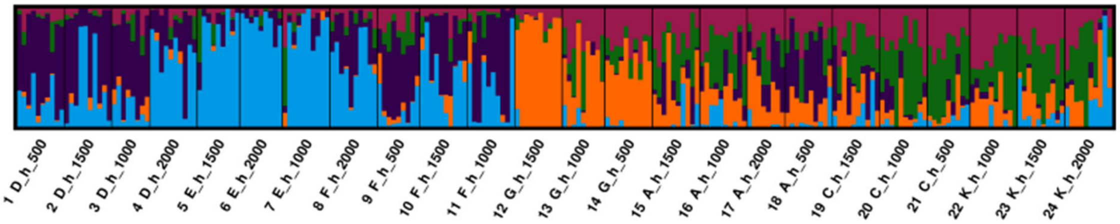

K = 6

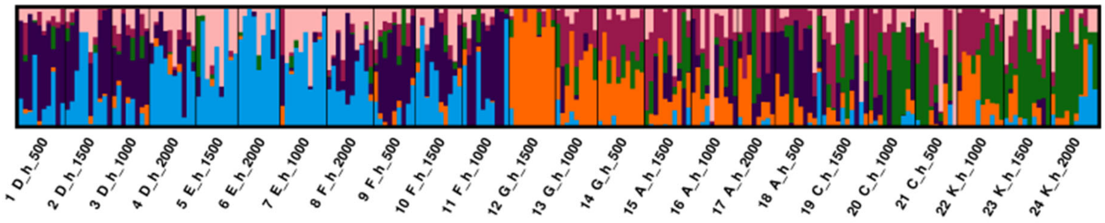

based on 761 supposedly selectively neutral SNPs

samples arranged in order of increasing altitude

K = 2

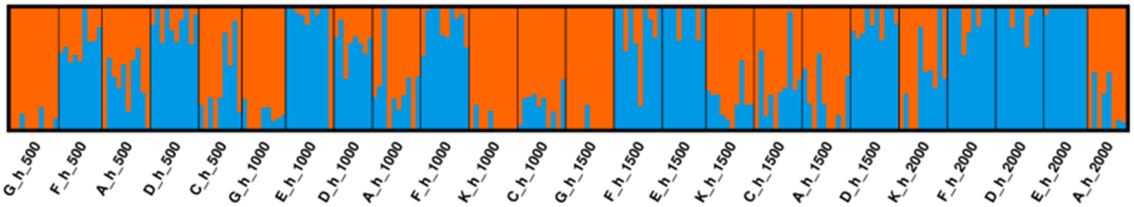

K = 3

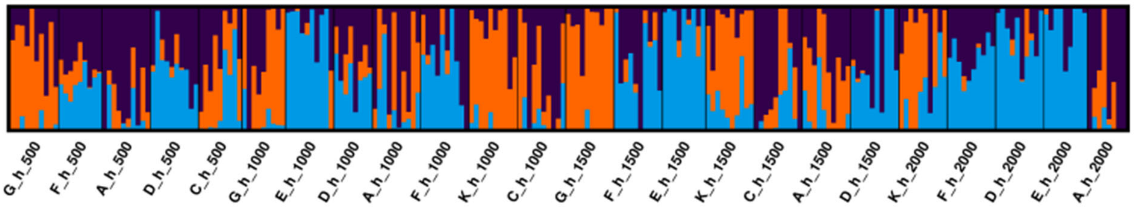

K = 4

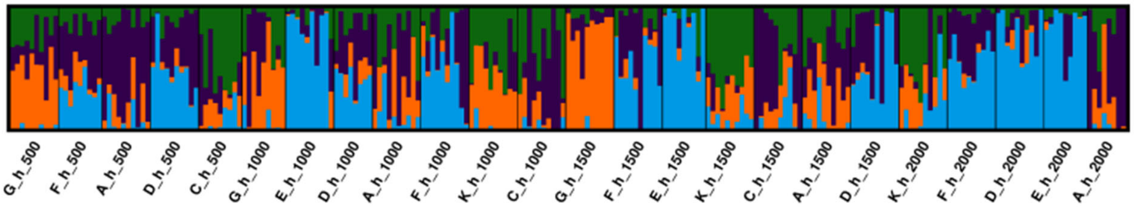

K = 5

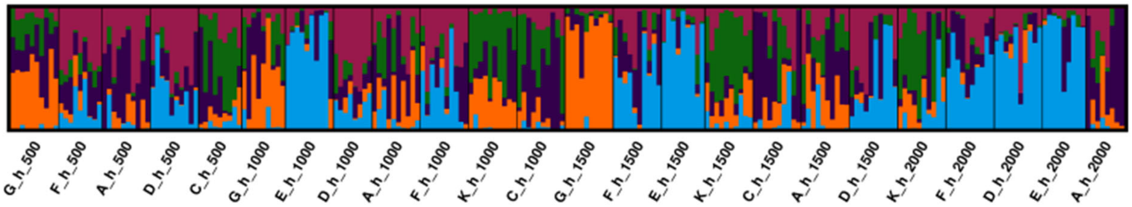

K = 6

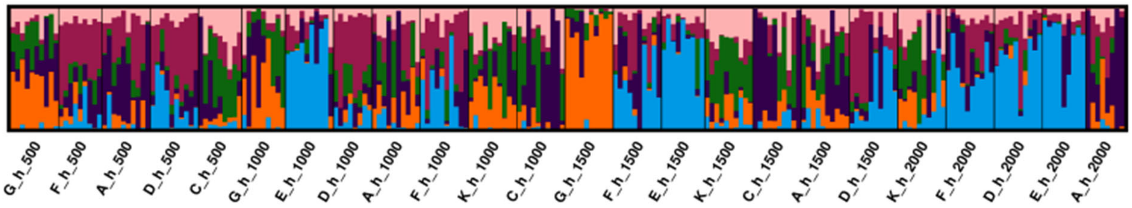

based on all 25,143 SNPs

samples arranged according to their geographic origin

$K = 2$

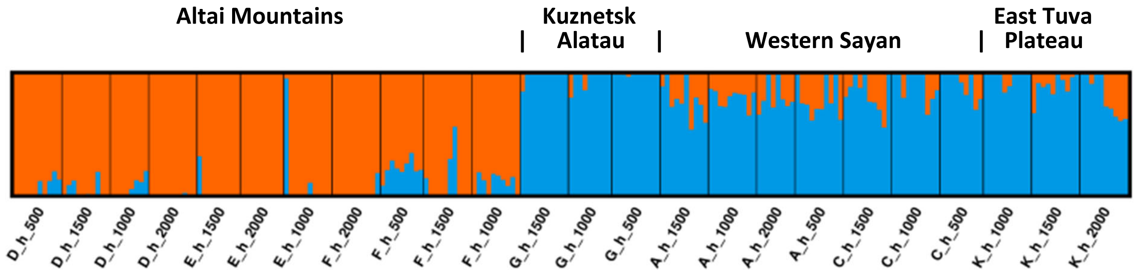

$K = 3$

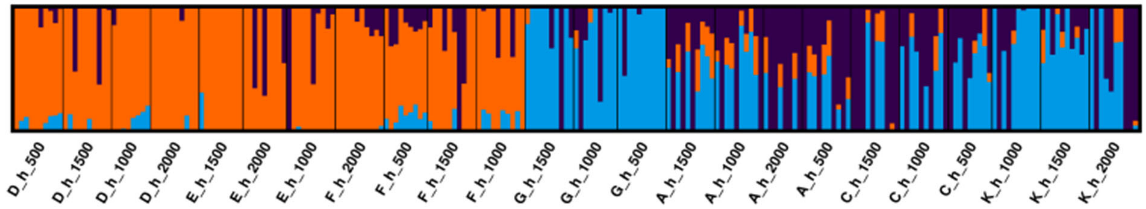

$K = 4$

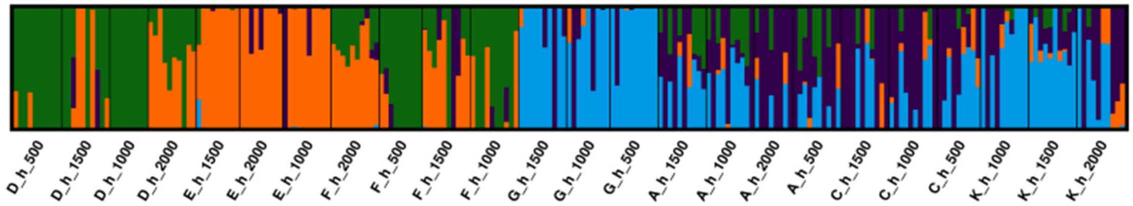

$K = 5$

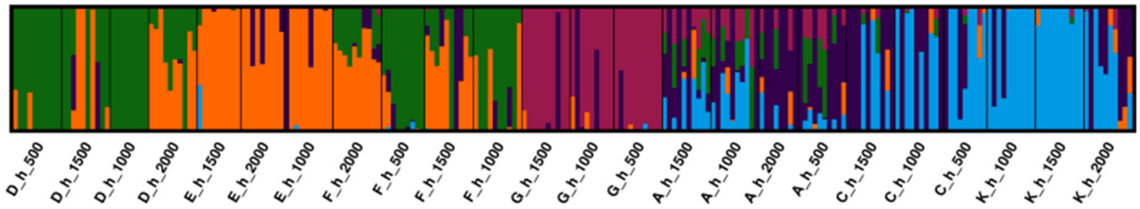

$K = 6$

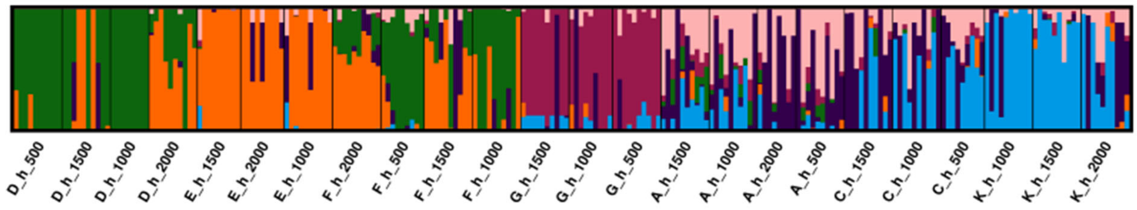

based on all 25,143 SNPs

arranged in order of increasing altitude

K = 2

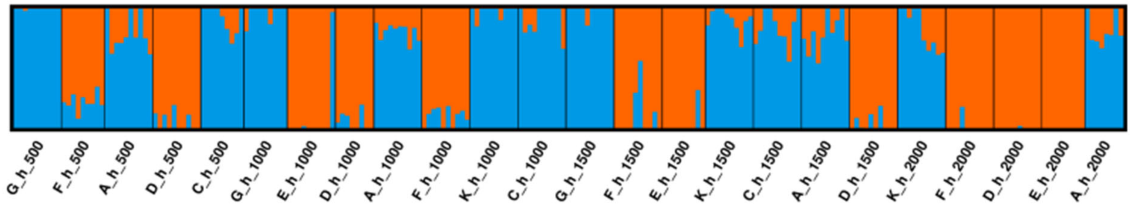

K = 3

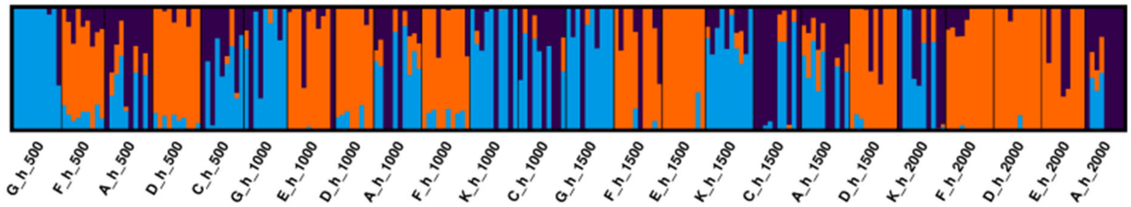

K = 4

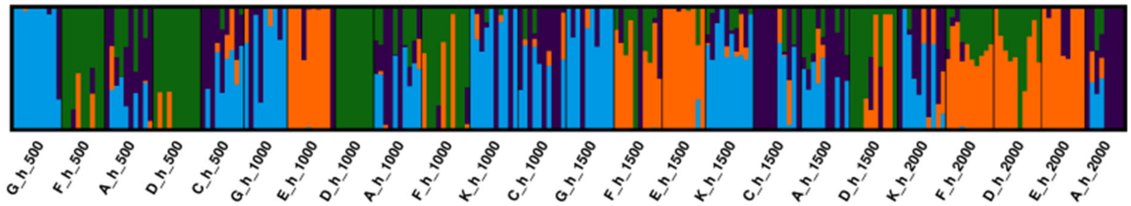

K = 5

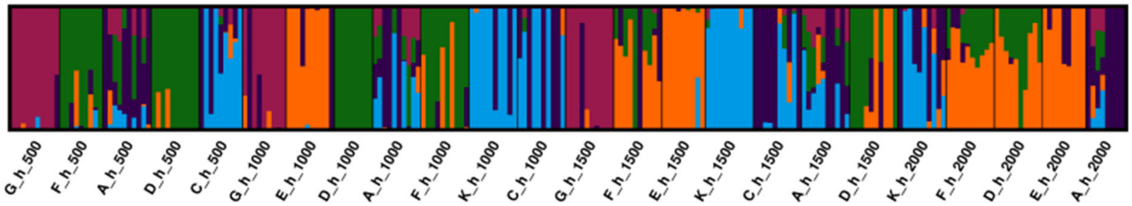

K = 6

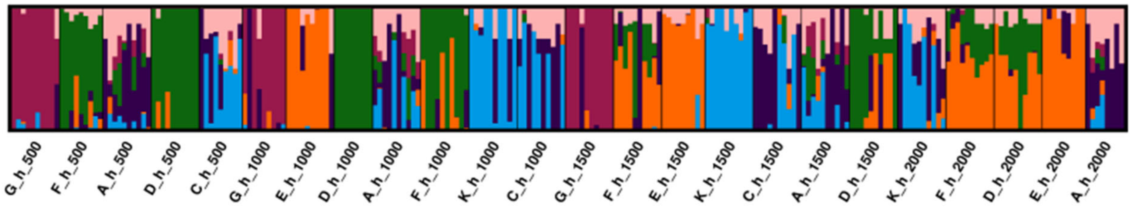

Based on 550 adaptive SNPs

samples arranged according to their geographic origin

K = 2

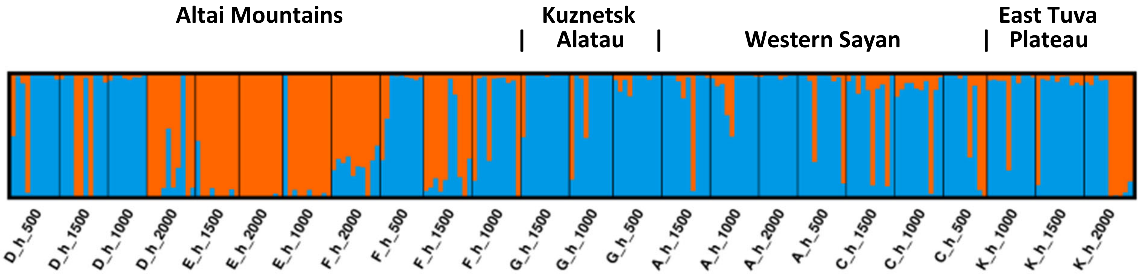

K = 3

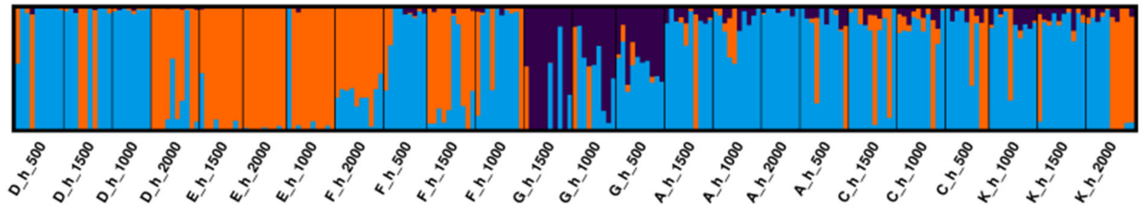

K = 4

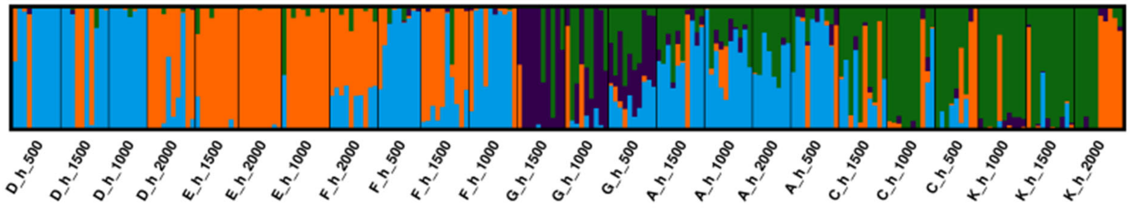

K = 5

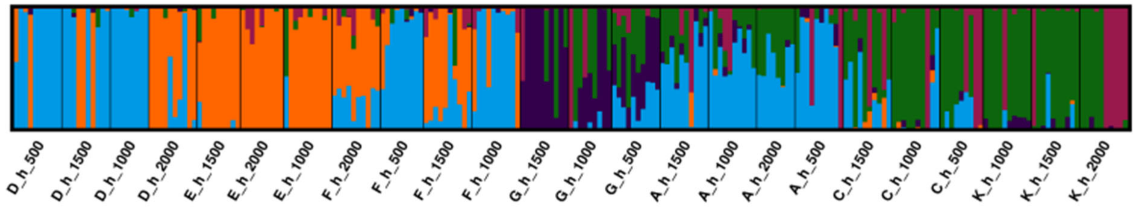

K = 6

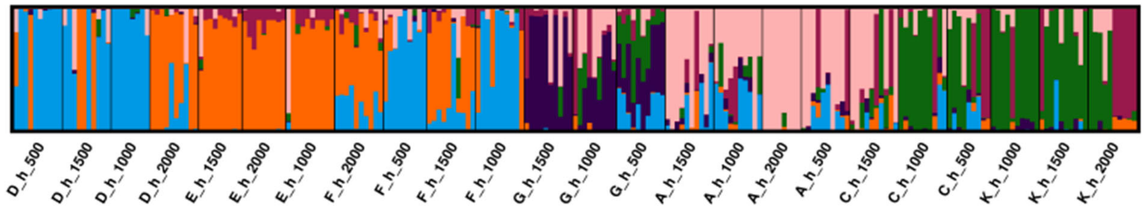

based on 550 adaptive SNPs

samples arranged in order of increasing altitude

K = 2

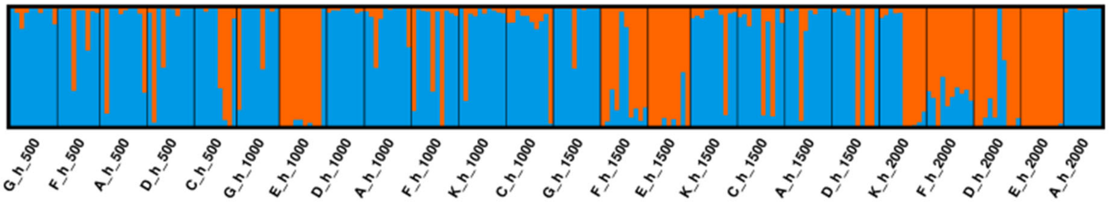

K = 3

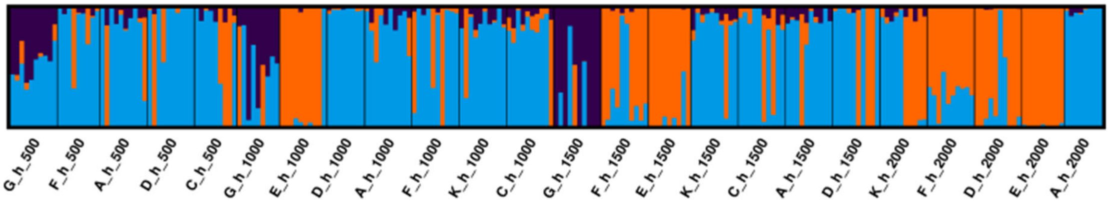

K = 4

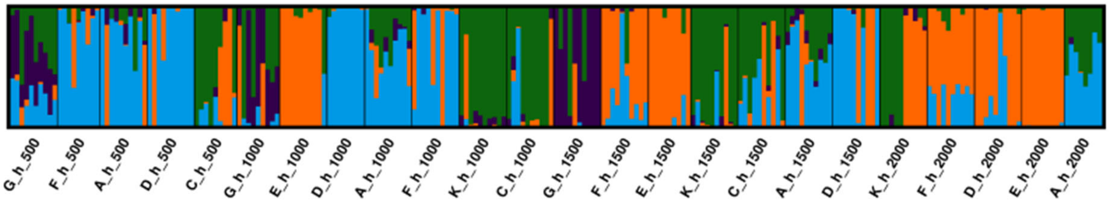

K = 5

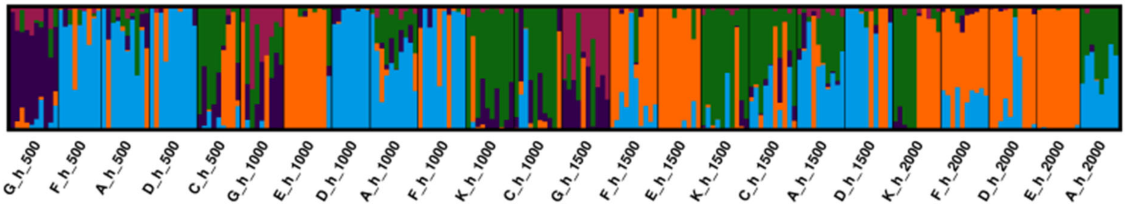

K = 6

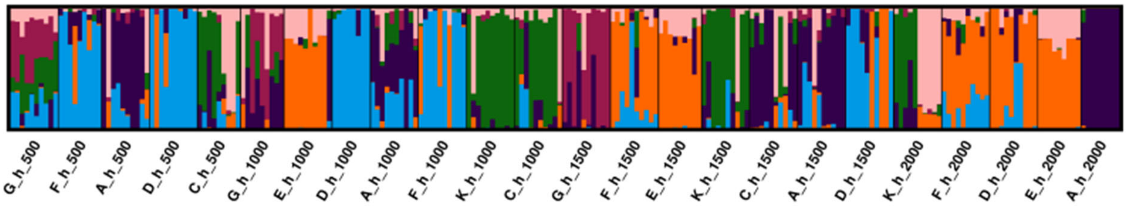

Figure S1. Admixture of each of the *K* clusters indicated by *K* different colors (Q-values) in the individual Siberian larch trees, representing 24 samples collected at different altitudes in four geographic regions and based on three different SNP datasets.
