# Supplementary material for: Genetic Adaptation of Siberian Larch (Larix sibirica Ledeb.) to High Altitudes
Source: Int J Mol Sci. 2023 Feb 25;24(5):4530. doi: 10.3390/ijms24054530 (PMC10003562; doi:10.3390/ijms24054530)
Supplement: Supplementary file 1 [file ijms-24-04530-s001.zip › Table S1. Mapping of the ddRADseq reads to the reference Siberian larch genome.pdf]

Table S1. Mapping of the ddRADseq reads to the reference Siberian larch genome.

Transect	Reads per sample, M	Unmapped reads, M	Aligned exactly one time, M	Aligned more than one time, M	Overall alignment rate
A	16.5 ± 9.9	0.690	1.378	14.432	96.22%
C	14.7 ± 13.2	0.423	1.313	13.032	97.22%
D	9.1 ± 3.4	0.176	0.698	8.241	98.08%
E	16.9 ± 10.5	0.948	1.469	14.512	94.59%
F	6.4 ± 2.3	0.148	0.487	5.835	97.60%
G	13.1 ± 5.1	0.257	1.116	11.775	98.02%
K	12.9 ± 6.02	0.374	1.092	11.493	97.22%
