# Supplementary material for: Genetic Adaptation of Siberian Larch (Larix sibirica Ledeb.) to High Altitudes
Source: Int J Mol Sci. 2023 Feb 25;24(5):4530. doi: 10.3390/ijms24054530 (PMC10003562; doi:10.3390/ijms24054530)
Supplement: Supplementary file 1 [file ijms-24-04530-s001.zip › Table S3. Summary of genetic variation parameters based on 761 neutral and 550 adaptive outlier SNPs_revised.pdf]

Table S3. Summary of genetic variation parameters based on 761 neutral and 550 adaptive outlier SNPs for each of 24 population samples of Siberian larch.

Region	Transect	Population sample	*N*	*PrA*	A_R	H_o	H_e	F_{IS}
				761 neutral SNPs				
Western Sayan Mountain	A	A_h_500	10	0	1.241 ± 0.014	0.041 ± 0.003	0.059 ± 0.004	0.213 ± 0.027***
		A_h_1000	10	0	1.297 ± 0.014	0.058 ± 0.004	0.068 ± 0.004	0.097 ± 0.018***
		A_h_1500	10	0	1.324 ± 0.014	0.063 ± 0.003	0.073 ± 0.004	0.086 ± 0.016***
		A_h_2000	8	0	1.235 ± 0.014	0.035 ± 0.003	0.056 ± 0.004	0.265 ± 0.030***
	C	C_h_500	9	0	1.286 ± 0.015	0.056 ± 0.004	0.071 ± 0.004	0.140 ± 0.022***
		C_h_1000	10	4	1.264 ± 0.014	0.039 ± 0.003	0.064 ± 0.004	0.279 ± 0.026***
		C_h_1500	10	0	1.239 ± 0.013	0.043 ± 0.003	0.055 ± 0.003	0.156 ± 0.024***
Altai Mountains	D	D_h_500	10	0	1.283 ± 0.013	0.066 ± 0.004	0.065 ± 0.004	-0.004 ± 0.011
		D_h_1000	8	0	1.279 ± 0.015	0.066 ± 0.004	0.065 ± 0.004	-0.009 ± 0.012
		D_h_1500	10	0	1.253 ± 0.013	0.059 ± 0.004	0.060 ± 0.004	0.009 ± 0.013
		D_h_2000	10	0	1.293 ± 0.013	0.065 ± 0.004	0.066 ± 0.004	0.013 ± 0.012
	E	E_h_1000	10	0	1.267 ± 0.013	0.051 ± 0.003	0.062 ± 0.004	0.114 ± 0.020***
		E_h_1500	9	0	1.295 ± 0.014	0.063 ± 0.004	0.070 ± 0.004	0.073 ± 0.017**
		E_h_2000	9	0	1.266 ± 0.015	0.053 ± 0.004	0.066 ± 0.004	0.126 ± 0.021***
	F	F_h_500	9	0	1.285 ± 0.014	0.068 ± 0.004	0.066 ± 0.004	-0.020 ± 0.011
		F_h_1000	10	0	1.268 ± 0.013	0.062 ± 0.004	0.062 ± 0.004	-0.009 ± 0.010
		F_h_1500	10	0	1.263 ± 0.013	0.059 ± 0.004	0.059 ± 0.003	0.004 ± 0.012
		F_h_2000	10	0	1.250 ± 0.013	0.060 ± 0.004	0.057 ± 0.004	-0.024 ± 0.011
Kuznetsk Alatau	G	G_h_500	10	0	1.290 ± 0.014	0.061 ± 0.004	0.069 ± 0.004	0.075 ± 0.017**
		G_h_1000	9	0	1.323 ± 0.015	0.067 ± 0.004	0.078 ± 0.004	0.084 ± 0.018**
		G_h_1500	10	0	1.331 ± 0.014	0.072 ± 0.004	0.084 ± 0.004	0.097 ± 0.017***
East Tuva Highlands	K	K_h_1000	10	5	1.299 ± 0.014	0.065 ± 0.004	0.072 ± 0.004	0.060 ± 0.015*
		K_h_1500	10	0	1.327 ± 0.014	0.064 ± 0.004	0.076 ± 0.004	0.104 ± 0.017***
		K_h_2000	10	0	1.304 ± 0.015	0.057 ± 0.003	0.073 ± 0.004	0.148 ± 0.021***
	Mean		9.6	0.375	1.282 ± 0.006	0.058 ± 0.002	0.067 ± 0.001	0.087 ± 0.017
				550 adaptive outlier SNPs				
Western Sayan Mountain	A	A_h_500	10	0	1.352 ± 0.019	0.063 ± 0.006	0.109 ± 0.007	0.351 ± 0.032***
		A_h_1000	10	0	1.366 ± 0.018	0.093 ± 0.006	0.098 ± 0.006	0.052 ± 0.020
		A_h_1500	10	0	1.425 ± 0.018	0.093 ± 0.007	0.114 ± 0.006	0.174 ± 0.024***
		A_h_2000	8	0	1.216 ± 0.016	0.056 ± 0.006	0.063 ± 0.006	0.100 ± 0.037
	C	C_h_500	9	0	1.341 ± 0.019	0.075 ± 0.006	0.115 ± 0.008	0.228 ± 0.029***
		C_h_1000	10	0	1.347 ± 0.018	0.060 ± 0.005	0.105 ± 0.007	0.324 ± 0.031***
		C_h_1500	10	0	1.349 ± 0.018	0.080 ± 0.007	0.111 ± 0.007	0.206 ± 0.029***
Altai Mountains	D	D_h_500	10	0	1.323 ± 0.018	0.105 ± 0.009	0.098 ± 0.006	0.009 ± 0.027
		D_h_1000	8	0	1.251 ± 0.017	0.083 ± 0.008	0.074 ± 0.006	-0.059 ± 0.028
		D_h_1500	10	0	1.346 ± 0.019	0.088 ± 0.008	0.120 ± 0.008	0.169 ± 0.034***
		D_h_2000	10	0	1.341 ± 0.018	0.111 ± 0.008	0.109 ± 0.007	0.016 ± 0.023
	E	E_h_1000	10	0	1.303 ± 0.018	0.069 ± 0.007	0.090 ± 0.006	0.269 ± 0.034***
		E_h_1500	9	0	1.312 ± 0.017	0.090 ± 0.007	0.086 ± 0.006	-0.016 ± 0.019
		E_h_2000	9	0	1.206 ± 0.016	0.064 ± 0.007	0.066 ± 0.006	0.022 ± 0.029
	F	F_h_500	9	0	1.347 ± 0.018	0.109 ± 0.008	0.098 ± 0.006	-0.038 ± 0.023
		F_h_1000	10	0	1.313 ± 0.018	0.091 ± 0.008	0.105 ± 0.007	0.085 ± 0.030
		F_h_1500	10	0	1.321 ± 0.018	0.098 ± 0.008	0.108 ± 0.007	0.077 ± 0.027
		F_h_2000	10	0	1.314 ± 0.018	0.139 ± 0.011	0.104 ± 0.007	-0.204 ± 0.02***

Region	Transect	Population sample	*N*	*PrA*	A_R	H_o	H_e	F_{IS}
Kuznetsk Alatau	G	G_h_500	10	0	1.487 ± 0.018	0.133 ± 0.007	0.139 ± 0.007	0.041 ± 0.017
		G_h_1000	9	0	1.646 ± 0.018	0.154 ± 0.007	0.200 ± 0.007	0.170 ± 0.022***
		G_h_1500	10	21	1.755 ± 0.016	0.203 ± 0.008	0.252 ± 0.007	0.206 ± 0.021***
East Tuva Highlands	K	K_h_1000	10	0	1.377 ± 0.018	0.077 ± 0.006	0.111 ± 0.007	0.230 ± 0.028***
		K_h_1500	10	0	1.37 ± 0.018	0.078 ± 0.006	0.107 ± 0.006	0.226 ± 0.029***
		K_h_2000	10	0	1.387 ± 0.019	0.070 ± 0.006	0.129 ± 0.008	0.292 ± 0.031***
	Mean		9.6	0.994	1.364 ± 0.024	0.097 ± 0.007	0.114 ± 0.008	0.113 ± 0.028

N - number of trees, *PrA* - the number of private alleles, A_R - allelic richness, H_o - observed heterozygosity, H_e - expected heterozygosity, F_{IS} - fixation index; * $P < 0.05$, ** $P < 0.01$, *** $P < 0.001$
