# Supplementary figures and images for: Genetic Adaptation of Siberian Larch (Larix sibirica Ledeb.) to High Altitudes
Source: Int J Mol Sci. 2023 Feb 25;24(5):4530. doi: 10.3390/ijms24054530 (PMC10003562; doi:10.3390/ijms24054530)

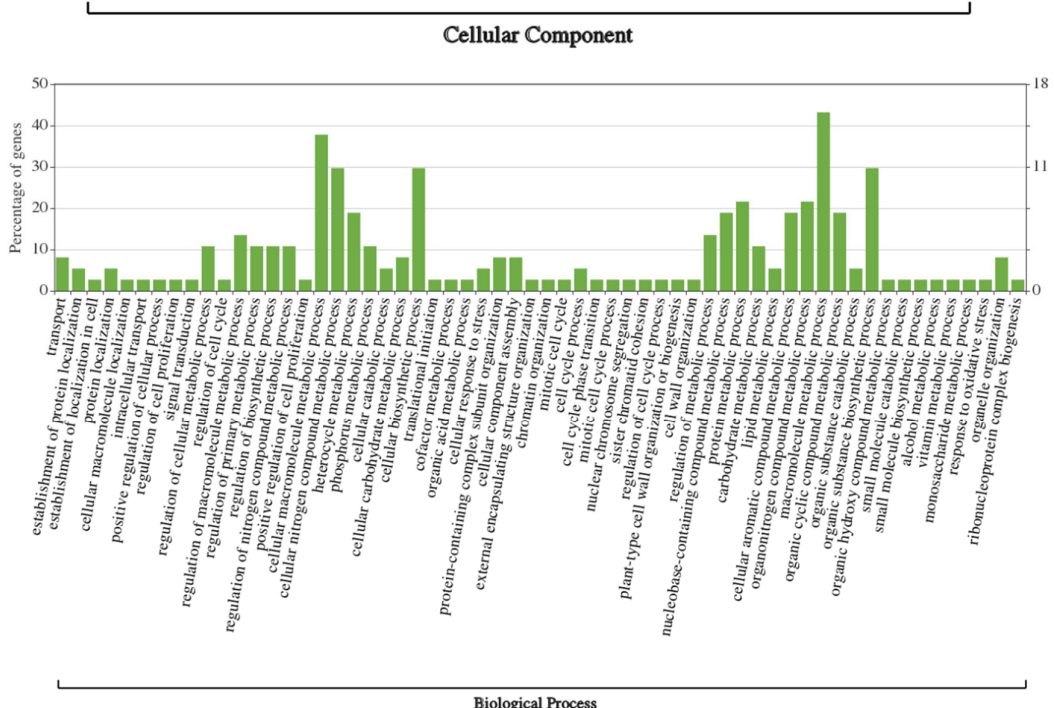

Number of genes

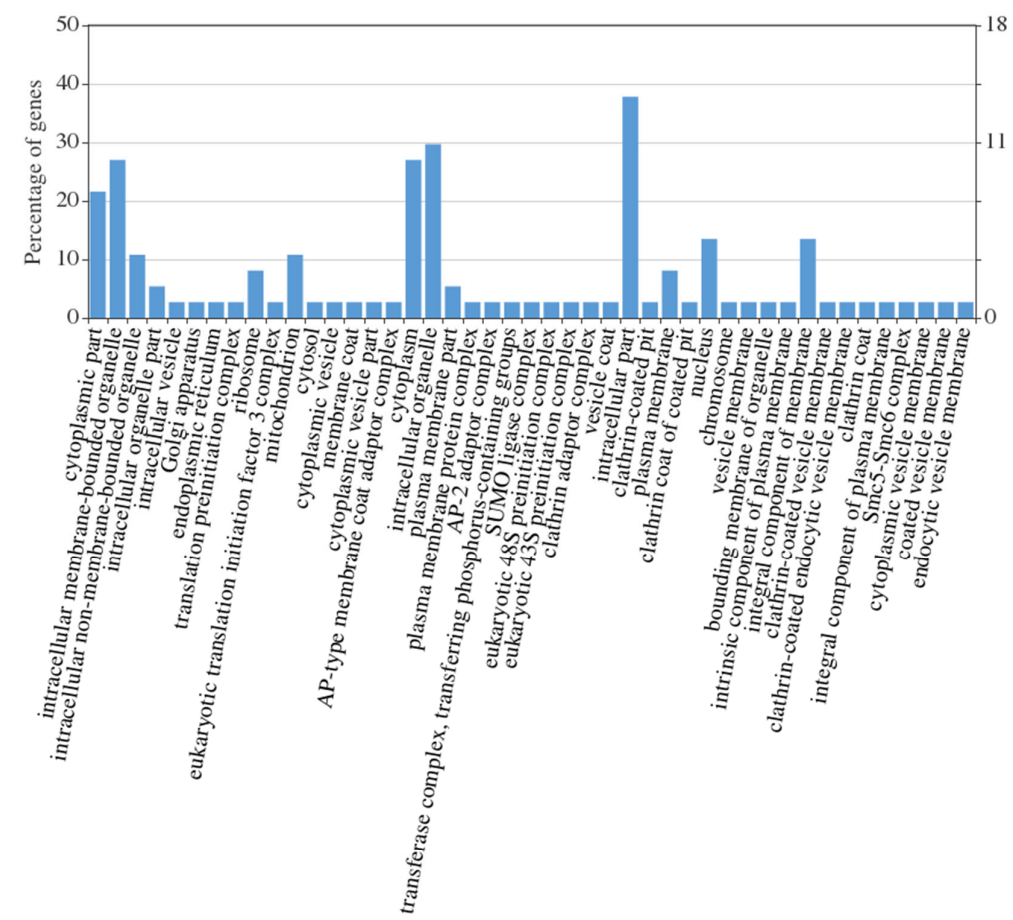

Number of genes

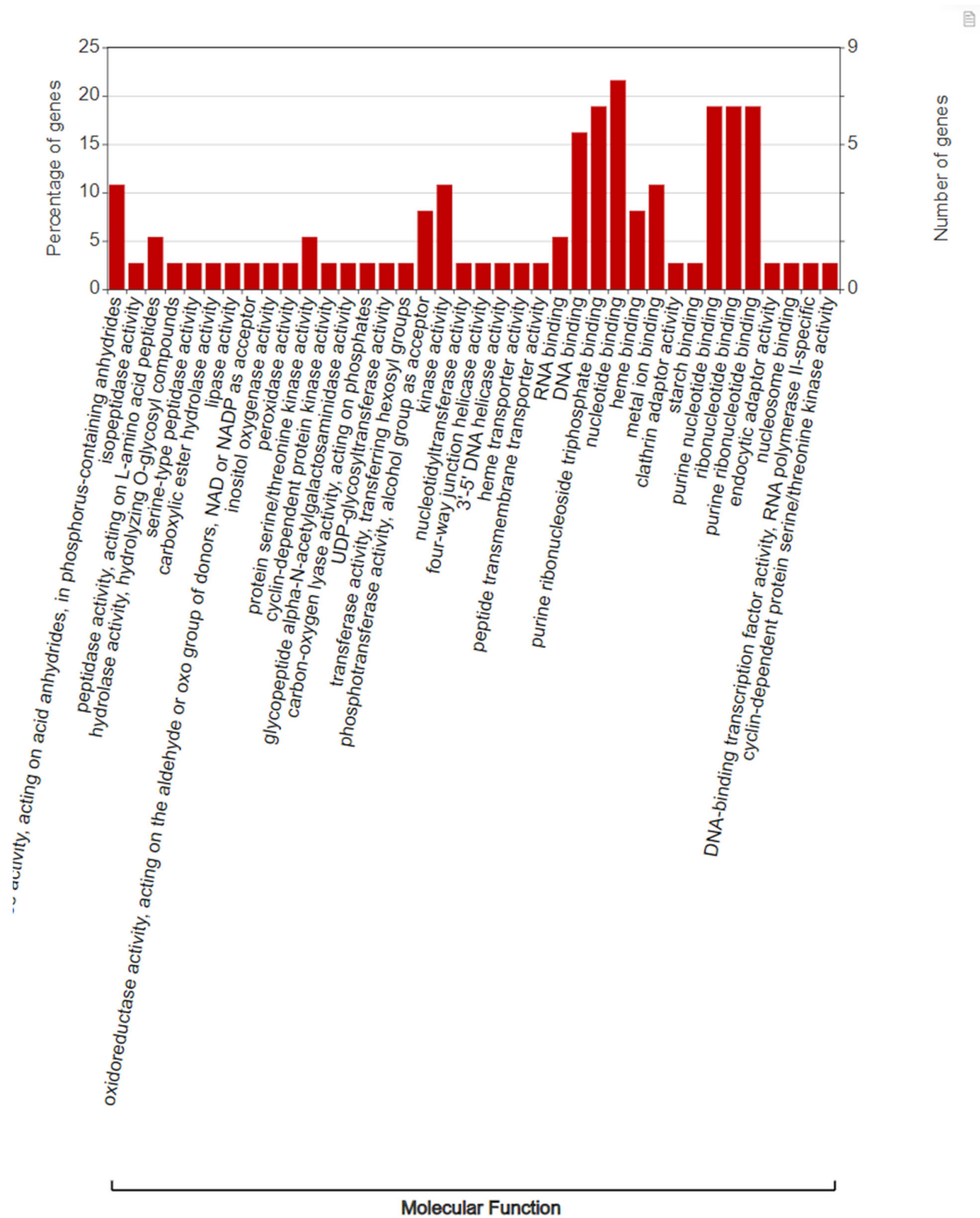

Figure S2. Gene ontology (GO) terms.

Supplement: Supplementary file 1 [file ijms-24-04530-s001.zip › Figure S2. Gene ontology (GO) terms.pdf]
